# Motivation and Pleasure Domain Links to Social Function in College Students: A Network Analysis

**DOI:** 10.1002/pchj.70001

**Published:** 2025-02-24

**Authors:** Hui‐xin Hu, Ling‐ling Wang, Yi‐Jing Zhang, Han‐xue Yang, Yun‐ru Wang, Yi Wang, Simon S. Y. Lui, Raymond C. K. Chan

**Affiliations:** ^1^ Neuropsychology and Applied Cognitive Neuroscience Laboratory, CAS Key Laboratory of Mental Health Institute of Psychology, Chinese Academy of Sciences Beijing China; ^2^ Department of Psychology University of Chinese Academy of Sciences Beijing China; ^3^ Department of Psychology, School of Humanities and Social Sciences Beijing Forestry University Beijing China; ^4^ School of Psychology Shanghai Normal University Shanghai China; ^5^ Faculty of Psychology Beijing Normal University Beijing China; ^6^ School of Psychology Beijing Language and Culture University Beijing China; ^7^ Department of Psychiatry, School of Clinical Medicine The University of Hong Kong, Hong Kong Special Administrative Region China

**Keywords:** alexithymia, college students, motivation and pleasure, network analysis, social functioning

## Abstract

Evidence suggests that the motivation and pleasure deficit of negative symptoms determines the social functioning in patients with schizophrenia spectrum disorder. Alexithymia is defined as the diminished ability to identify and describe emotion feelings, and influences patients' social functioning. However, little is known regarding the relationship between motivation and pleasure, alexithymia, and social functioning in nonclinical populations. This network analysis study aimed to investigate the interactions between motivation and pleasure, alexithymia and social functioning in a sample of 2889 college students. The flow network and item‐level regularized partial correlation network were constructed. Centrality estimation and relative importance metrics were also estimated. The network structures between subgroups with high and low social anhedonia were compared. Our resultant networks showed that the motivation factor was closely connected with social functioning. The relative importance analysis found that, among other nodes, the motivation factor accounted for the highest proportion of variance of social functioning in the nonclinical sample. Although the two subgroups with high and low social anhedonia differed significantly in network structures, they generally shared a similar edge structure. The two subgroups only exhibited significant difference in the connection between the social pleasure factor and recreation/work pleasure factor of the motivation and pleasure. Our findings supported the important role of the motivation factor in determining social functioning in nonclinical population.

## 1 Introduction

Psychosis is a continuous phenotype (van Os et al. 2009), and schizophrenia lies at the extreme of the schizophrenia spectrum. Negative symptoms comprise the motivation and pleasure (MAP) factor (i.e., asociality, anhedonia, and avolition), and the expressivity (EXP) factor (i.e., blunted affect and alogia) in schizophrenia patients (Chan et al. 2015; Kirkpatrick et al. 2006; Kring et al. 2013). Nonclinical samples along the schizophrenia spectrum can exhibit subclinical features of schizophrenia, including negative symptoms (Kaiser et al. 2011; Sauvé et al. 2019). For instance, schizotypy refers to the latent personality construct which contributes to the liability of schizophrenia (Cohen et al. 2015; Grant et al. 2018). Evidence suggests that people with high levels of schizotypal features (Wang et al. 2014; Xie et al. 2018) and the clinical high‐risk (prodromal) population (Piskulic et al. 2012) showed subclinical negative symptoms, which may also predict the risk of developing clinical psychosis (Gooding et al. 2005). The MAP and EXP factors of negative symptoms have also been demonstrated in nonclinical populations of the psychosis spectrum (Xie et al. 2018).

Evidence suggested that the MAP deficits of negative symptoms in particular play a central role in determining schizophrenia patients' social functioning (Chang et al. 2020; Fervaha et al. 2015; Hu et al. 2022). In nonclinical populations, evidence suggested that anhedonia is negatively associated with social functioning (Chun et al. 2020; Wang, Neumann, et al. 2013), but limited research has been conducted to examine the relationship between motivation and social functioning. Motivation is a key determinant of social functioning in schizophrenia patients (Fervaha et al. 2015; Hu et al. 2022) and people at‐risk of developing psychosis (Fervaha et al. 2014; Gupta et al. 2021). However, it remains unclear how MAP deficits would interact with social functioning in nonclinical populations.

Alexithymia refers to the diminished ability to identify and describe emotion feelings (Taylor et al. 1991), and it has been found in schizophrenia patients (O'Driscoll et al. 2014; Van't Wout et al. 2007; Yi et al. 2023) and people with schizotypal traits (Aaron et al. 2015; Seghers et al. 2011; Yang et al. 2020). Interestingly, alexithymia is reported to predict clinical (Kimhy et al. 2012; Ospina et al. 2019) and subclinical (Kimhy et al. 2016) individuals' social functioning. On the other hand, the relationship between alexithymia and negative symptoms in schizophrenia patients remains inconclusive. For instance, several studies reported that alexithymia was positively correlated with negative symptoms (Demirkol et al. 2019; Van't Wout et al. 2007), but some failed to demonstrate such relationship (Fogley et al. 2014; Todarello et al. 2005).

Previous studies using correlational and regression analyses (Chun et al. 2020; Wang, Neumann, et al. 2013) might not have adequately accounted for the complex interactions among MAP deficits, alexithymia, and social functioning in nonclinical populations. The network approach has become popular in the psychopathology research, which conceptualized mental disorders as the complex network of mutually interacting symptoms (Borsboom et al. 2018; Bringmann et al. 2022; Roefs et al. 2022). This useful statistical method could address complex interactions between variables, and estimate centrality indices of nodes in the network (Borsboom 2017; Borsboom and Cramer 2013), which may indicate suitable intervention targets. Previous studies have used network analysis to unveil the complex interaction between clinical symptoms and social functioning in schizophrenia patients (Chang et al. 2020; Galderisi et al. 2018; Hu et al. 2022). The MAP factor was found to be the most central role in the networks of clinical schizophrenia patients (Chang et al. 2020; Hu et al. 2022). Among the studies on nonclinical samples, few have applied network analysis. For instance, a recent network analysis study investigated the interrelationship of schizotypy traits (Polner et al. 2021), and another study investigated the complex interactions between schizotypy traits and alexithymia (Yang et al. 2020). To our knowledge, no network analysis study has been employed for examining the complex interactions between the MAP factor, alexithymia, and social functioning in a nonclinical sample.

This study aimed to investigate the interactions between MAP deficits, alexithymia and social functioning in a sample of college students using regularized partial correlation network analysis. We also aimed to identify nodes which might play influential role, using centrality indices and relative importance metrics. Additionally, social anhedonia (SA) refers to a trait‐like reduced ability to experience pleasure during social activities (Blanchard et al. 2011; Cohen et al. 2015). People with high levels of SA have high risk for developing schizophrenia‐spectrum disorders (Cohen et al. 2020; Gooding et al. 2005) and show similar but less severe MAP deficits and social functioning impairments observed in patients with schizophrenia (Cohen et al. 2020; Xie et al. 2018). In this study, we compared the differences in network structure of these variables between the subgroups with high and low levels of SA. Moreover, we also investigated the gender differences in the network structure. Based on earlier findings (Chang et al. 2020; Fervaha et al. 2014; Hu et al. 2022), we hypothesized that the MAP factor, particularly motivation, play the central role to determine social functioning.

## 2 Materials and Methods

### 2.1 Participants

Our sample comprised 3901 college students aged 18–30, recruited from 28 regions in Chinese mainland via online advertisements. All participants completed a set of online self‐report questionnaires, measuring MAP factors, alexithymia, and social functioning. After excluding 814 (196 males) ineligible subjects (see Supporting Information Methods for the exclusion criteria), our final sample comprised 2889 eligible participants. The sample size was sufficient because a network of 20 nodes or less would only need 250–350 participants (Constantin and Cramer 2022). This study was approved by the Ethics Committee of the Institute of Psychology, the Chinese Academy of Sciences (Ethics number: H20041). All participants provided informed written consent online. Each participant was given 40 RMB (around US $6) as incentive after completion of this study.

### 2.2 Measures

#### 2.2.1 The Motivation and Pleasure Scale—Self‐Report (MAP‐SR)

It (Llerena et al. 2013; Wang et al. 2020) was used to assess motivation and pleasure factor of negative symptoms. The MAP‐SR has 15 items and four factors. All items were rated on a five‐point Likert format. Higher scores indicate greater impairments in motivation and pleasure factors. The Chinese version of MAP‐SR has good internal consistency, test–retest reliability and validity (Wang et al. 2020).

#### 2.2.2 The Toronto Alexithymia Scale—20 (TAS‐20)

It (Bagby et al. 1994; Zhu et al. 2007) was used to assess alexithymia. This scale comprises 20 items and three factors, namely difficulty in identifying feelings (DIF), difficulty in describing feelings (DDF), and externally oriented cognitive style thinking (EOT). Higher scores indicate higher alexithymia. The Chinese version of TAS‐20 has good reliability and validity (Zhu et al. 2007).

#### 2.2.3 The Chinese version of the First‐Episode Social Functioning Scale (FESFS)

It (Lecomte et al. 2005; Lecomte et al. 2014; Wang, Yeh, et al. 2013) was used to assess the perceived ability of social functioning. The Chinese version of FESFS comprises 27 items and six subscales, including Living skills, Interpersonal, Intimacy, Family and friends, School, and Balance. Higher scores indicate greater social functioning. FESFS has good reliability and construct validity among Chinese college students (Wang, Yeh, et al. 2013), and has been used to evaluate social functioning in nonclinical samples (Shi et al. 2017; Wang et al. 2018).

#### 2.2.4 The Revised Chapman Social Anhedonia Scale (CSAS)

It (Chan et al. 2012; Chapman et al. 1976) was used to measure trait SA. The CSAS comprises 40 “true‐false” items. Higher scores indicate more severe SA. The CSAS has good reliability and construct validity (Chan et al. 2012).

### 2.3 Data Analysis

Descriptive analyses were performed using the SPSS (Version 22.0) (IBM 2013). Our final sample (*n* = 2889) did not have any non‐response or missing data. Tables S1 and S2 show the skewness, kurtosis, and psychometric properties (i.e., internal consistency reliability and dimensionality) of all measures, including the factors of the MAP‐SR, the TAS, and the FESFS. Gender differences in demographics and self‐report measures were examined using independent‐sample *t* test and chi‐square test.

Network estimation and visualization, centrality indices and relative importance analyses were conducted using the R statistical software (version 4.2.3) (R Core Team 2023) (see Supporting Information for the R script). First, to elucidate the effects of MAP and TAS factors on social functioning, a “flow network” was estimated using the R‐packages *qgraph* version 1.9.5 (Epskamp et al. 2012), and *bootnet* version 1.5.3 (Epskamp et al. 2018). Eight variables (the FESFS total score, the MAP‐SR factors, the TAS factors) were included as nodes in the flow network. The edge between two nodes of the network was calculated as a partial correlation between two nodes, while controlling for all other nodes in the network. The graphical Least Absolute Shrinkage and Selection Operator (gLASSO) regularization technique (Friedman et al. 2008) in combination with the Extended Bayesian Information Criterion (EBIC) model selection (Chen and Chen 2008; Foygel and Drton 2010) was applied to obtain the optimal network. The flow network was visualized using the Reingold–Tilford graph layout algorithm (Reingold and Tilford 1981). To identify which nodes play a central role in the network, the centrality indices (i.e., strength, closeness, betweenness and expected influence [EI]) (Borsboom and Cramer 2013; Robinaugh et al. 2016) for each node were estimated using the R‐packages *qgraph* (Epskamp et al. 2012). The bridge strength and bridge EI were also estimated to measure how well the nodes would connect to the other domains of interest regardless of their own cluster membership (Jones et al. 2021). Moreover, the predictability (Haslbeck and Fried 2017; Haslbeck and Waldorp 2020) for each node were calculated using the R‐packages *mgm* (Haslbeck and Waldorp 2020). The stability and accuracy of the estimated network were evaluated using the R‐package *Bootnet*, according to Epskamp's guidance (Epskamp et al. 2018) (see Supporting Information Methods for details).

Apart from “flow network” which reflected the direct effect of each variable on social functioning, we further conducted “relative importance analysis” using the R‐package *relaimpo* version 2.2–6 (Grömping 2007) to estimate the quantitative contribution of each variable to social functioning, which accounted for both the direct effect of a variable and its indirect effect from other variables. A univariate linear regression model was conducted, with the FESFS total score as the dependent variable and the remaining variables as the independent variables. The explained variance (*R* ^2^) of social functioning was then partitioned into the quantitative contribution of each variable using the *lmg* metric (Lindeman et al. 1980).

To further explore how each item of the MAP and TAS would interact with specific aspects of social functioning, we constructed an “item‐level” network, using each item of all the scales as nodes. According to empirical estimates, it takes at least 20–30 participants to include one node in the network (Epskamp et al. 2018; Hair et al. 2018). Our sample size was sufficient to estimate such “item‐level” network. The estimation procedure was identical to the flow network analysis above. The resultant network was visualized using the Fruchterman–Reingold algorithm (Fruchterman and Reingold 1991).

Finally, to explore whether the network structure would differ between the subgroups with high versus low levels of SA, we defined participants who scored on the top 10th percentile of the CSAS (> 20) as the “high‐SA” subgroup (*n* = 246), and those scored below the mean CSAS (< 11) as the “low‐SA” subgroup (*n* = 1612). Network Comparison Test (NCT) was conducted using the R‐package NCT version 2.2.1 (Van Borkulo et al. 2019), with 1000 iterations. Likewise, we conducted NCT to examine gender difference in network invariance and global strength (see Supporting Information Results for details).

## 3 Results

### 3.1 Descriptive Statistics and Gender Effect

Table 1 shows the sample characteristics. Although male participants were significantly older in age (*p* = 0.002) and had higher education level (*p* = 0.006) than female participants, such differences in age and education were small (less than half a year). Regarding the MAP, male participants reported lower social pleasure (*p* = 0.004) but higher motivation and efforts to engage in activities (*p* = 0.007) than female counterparts. No gender difference was found in recreational/work pleasure, and feelings about relationships (*p*'s > 0.05). Regarding the TAS, male participants self‐reported significantly more difficulties in describing their feelings than female participants (*p* = 0.010). No significant gender difference in other two TAS factors (*p*'s > 0.05) was found.

**TABLE 1:** Demographic information and self‐reported questionnaires of all participants [mean (SD)].

	Total (*n* = 2889)	Male (*n* = 607)	Female (*n* = 2282)	*t* (df = 2887)	*p*	Cohen's *d*	95% CIs of Cohen's *d*
Age (years)	20.28 (1.81)	20.50 (2.03)	20.23 (1.74)	**3.056**	**0.002**	**0.140**	**[0.050, 0.229]**
Length of education (years)^a^	14.32 (1.69)	14.52 (1.88)	14.27 (1.63)	**2.741**	**0.006**	**0.134**	**[0.038, 0.230]**
Ethnicity, *n* (%)							
Han	2645 (91.55)	558 (91.93)	2087 (91.45)	—	—	—	—
Non‐Han	244 (8.45)	49 (8.07)	195 (8.55)	—	—	—	—
Socioeconomic status							
Father's education (years)^b^	10.15 (3.63)	10.31 (3.61)	10.11 (3.63)	1.166	0.244	0.054	[−0.034, 0.144]
Mother's education (years)^c^	9.28 (3.89)	9.36 (3.83)	9.26 (3.91)	0.567	0.571	0.026	[−0.064, 0.116]
Monthly household income, *n* (%)							
≦ 2500 RMB	211 (7.30)	31 (5.11)	180 (7.89)	—	—	—	—
2501–5000 RMB	823 (28.49)	140 (23.06)	683 (29.93)	—	—	—	—
5001–10,000 RMB	1073 (37.14)	235 (38.71)	838 (36.72)	—	—	—	—
10,001–20,000 RMB	579 (20.04)	148 (24.38)	431 (18.89)	—	—	—	—
≧ 20,001 RMB	203 (7.03)	53 (8.73)	150 (6.57)	—	—	—	—
CSAS score	10.70 (6.42)	10.96 (6.51)	10.63 (6.39)	1.132	0.258	0.052	[−0.038, 0.141]
MAP‐SR total score	20.34 (9.18)	20.28 (9.59)	20.36 (9.07)	−0.179	0.858	−0.008	[−0.098, 0.081]
Social pleasure	3.18 (2.33)	3.42 (2.38)	3.12 (2.31)	**2.880**	**0.004**	**0.132**	**[0.042, 0.221]**
Recreational or work pleasure	3.57 (2.43)	3.74 (2.52)	3.53 (2.40)	1.954	0.051	0.089	[−0.000, 0.179]
Feelings about relationships	3.84 (2.56)	3.78 (2.67)	3.86 (2.53)	−0.688	0.491	−0.031	[−0.121, 0.058]
Motivation	9.74 (4.22)	9.34 (4.27)	9.85 (4.20)	**−2.683**	**0.007**	**−0.123**	**[−0.212, −0.033]**
TAS total score	53.22 (10.33)	53.71 (11.01)	53.10 (10.14)	1.239	0.216	0.057	[−0.033, 0.146]
Difficulty in identifying feelings (DIF)	18.11 (5.39)	18.30 (5.73)	18.06 (5.30)	0.938	0.349	0.043	[−0.047, 0.132]
Difficulty in describing feelings (DDF)	13.98 (3.77)	14.34 (3.98)	13.88 (3.71)	**2.568**	**0.010**	**0.117**	**[0.028, 0.207]**
Externally oriented cognitive style of thinking (EOT)	21.14 (3.28)	21.07 (3.52)	21.16 (3.21)	−0.565	0.572	−0.026	[−0.115, 0.064]
FESFS total score	80.94 (8.44)	82.44 (9.09)	80.54 (8.22)	**4.666**	**< 0.001**	**0.213**	**[0.123, 0.303]**
Interpersonal	18.85 (2.97)	19.36 (3.17)	18.71 (2.90)	**4.800**	**< 0.001**	**0.219**	**[0.130, 0.309]**
Family and friends	16.06 (2.10)	15.96 (2.16)	16.09 (2.08)	−1.341	0.180	−0.061	[−0.151, 0.028]
Living skills	12.78 (1.76)	12.81 (1.81)	12.77 (1.75)	0.450	0.653	0.021	[−0.069, 0.110]
Intimacy	9.01 (1.70)	9.73 (1.59)	8.81 (1.68)	**12.052**	**< 0.001**	**0.550**	**[0.460, 0.641]**
Balance	8.77 (1.37)	9.00 (1.42)	8.70 (1.35)	**4.762**	**< 0.001**	**0.218**	**[0.128, 0.307]**
School	15.47 (1.96)	15.58 (2.19)	15.45 (1.90)	1.345	0.179	0.061	[−0.028, 0.151]

*Note: p* < 0.05 are bold.

Abbreviations: CI = confidence interval; CSAS = The Chinese version of revised Chapman Social Anhedonia Scale; FESFS = The Chinese version of the First‐Episode Social Functioning Scale; MAP‐SR = The Motivation and Pleasure Scale—Self Report; SD = standard deviation; TAS = The Chinese version of 20‐item Toronto Alexithymia Scale.

^a^ Education information was missing for 363 participants, there were 2526 participants (532 males and 1994 females). The degree of freedom (df) of independent two sample t‐test for length of education was 2524.

^b^ Father's education information was missing for 53 participants, there were 2836 participants (600 males and 2236 females).

^c^ Mother's education information was missing for 47 participants, there were 2842 participants (600 males and 2242 females).

Regarding self‐perceived social functioning, male participants reported better performance than female participants in the interpersonal, intimacy, and balance subscales (*p*'s < 0.001), but not the other subscales (*p*'s > 0.05) of the FESFS.

### 3.2 The Flow Network

**FIGURE 1:**
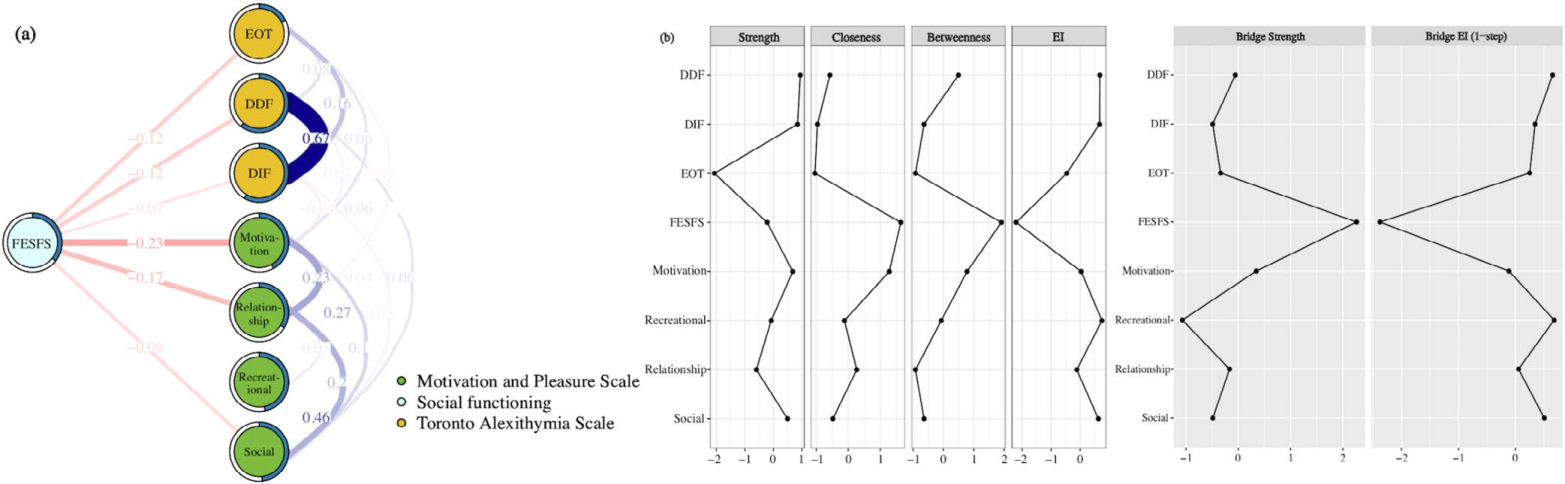
(a) Flow network of motivation and pleasure, alexithymia, and social functioning in college students (*n* = 2889). (b) The standardized centrality indices and bridge centrality indices of each node in the flow network. Each node in the network represents a variable. Each edge represents the regularized partial correlation between two nodes controlled for all other nodes. Blue edges represent positive associations, whereas red edges represent negative associations. Thicker edges (positive and negative) signify stronger partial correlations. The pie chart surrounding each node represents node predictability. The value of each edge represents the value of the regularized partial correlation coefficients. DDF = difficulty in describing feelings of TAS; DIF = difficulty in identifying feelings factor of TAS; EI = expected influence; EOT = Externally oriented cognitive style thinking of TAS; FESFS = The Chinese version of the First‐Episode Social Functioning Scale; MAP‐SR = The Motivation and Pleasure Scale—Self‐Report; Motivation = motivation and effort in engaging in activities factor of MAP‐SR; Recreational = recreational/work pleasure factor of MAP‐SR; Relationship = feelings about relationship factor of MAP‐SR; Social = social pleasure factor of MAP‐SR; TAS = The Chinese version of 20‐item Toronto Alexithymia Scale.

Figure 1a shows the flow network, which indicates the interactions of the MAP and TAS factors on social functioning. Regarding the edge‐weight accuracy and centrality stability, we found that the edge‐weight estimates were reliable and accurate, and all the centrality indices showed sufficient stability (see Supporting Information Results and Figures S1 and S2).

On inspection, we found that the “motivation” factor (edge value = −0.23) and the “feelings about relationship” factor (edge value = −0.17) were closely and negatively connected with social functioning. By contrast, nodes of the TAS showed relatively weak and negative connections with social functioning (edge values = −0.07 to −0.12). The bootstrapped difference test results for edge‐weights are shown in Figure S3.

Figure 1b shows the standardized centrality estimates of each node. The MAP “motivation” factor and “social pleasure” factor, and the TAS DIF and DDF factors demonstrated significantly higher node strength. Apart from social functioning, the MAP “motivation” factor showed significantly higher node closeness than most other nodes, indicating that this factor showed short mean distance to other nodes. Social functioning showed the highest betweenness (but not reaching statistical significance in the bootstrapped difference tests), indicating that social functioning might be important in linking all other nodes. The MAP “recreational/work pleasure” factor showed the highest EI, indicating that the sum of edge weights of the MAP “recreational/work pleasure” factor was highest in the network, but such result did not reach statistical significance in the bootstrapped difference tests (see Figure S4 for details). Regardless of the connections within their own cluster, social functioning showed the highest bridge strength, and the MAP “recreational/work pleasure” factor showed the highest bridge EI. Table S4 shows the *Z* scores of centrality indices, bridge centrality indices and predictability of each node in the flow network.

### 3.3 The Relative Importance Analysis

Table 2 summarizes the linear regression coefficient and the relative contribution of each variable to social functioning. The MAP and TAS factors together explained 35.71% of the variance of social functioning (*F*_(7,2881)_ = 228.563, *p* < 0.001). All variables significantly predicted social functioning (*p*'s < 0.001), except for the MAP “recreational/work pleasure” factor (*p* = 0.868). Notably, the MAP “motivation” factor accounted for 8.83% of the variance of social functioning, which was higher than the contribution from other variables (see Table S5 for bootstrapped confidence intervals of difference).

**TABLE 2:** Univariate linear regression and relative contribution of each variable to the total score of social functioning (*n* = 2889).

Independent variables	Unstandardized beta	Standardized beta	SE	*t* (df = 2881)	*p*	Explained *R* ^2^ (%)
MAP—Social pleasure	−0.379	−0.105	0.021	−5.047	**< 0.001**	4.529
MAP—Recreational or work pleasure	0.012	0.003	0.021	0.166	0.868	2.857
MAP—Feelings about relationship	−0.560	−0.170	0.018	−9.380	**< 0.001**	6.401
MAP—Motivation	−0.490	−0.245	0.019	−12.789	**< 0.001**	**8.827**
TAS—DIF	−0.168	−0.108	0.023	−4.600	**< 0.001**	4.453
TAS—DDF	−0.307	−0.137	0.024	−5.781	**< 0.001**	5.588
TAS—EOT	−0.287	−0.111	0.016	−6.813	**< 0.001**	3.051

*Note: p* < 0.05 are bold.

Abbreviations: DDF = difficulty in describing feelings of TAS; DIF = difficulty in identifying feelings of TAS; EOT = externally oriented cognitive style of thinking of TAS; MAP = The Motivation and Pleasure Scale—Self Report; SE = standard error of standardized beta; TAS = The Chinese version of 20‐item Toronto Alexithymia Scale.

### 3.4 Network Structure at the Item‐Level

To further investigate how the specific items of the MAP and the TAS would be interconnected with the aspects of social functioning, we constructed the item‐level network. We found a relatively high edge‐weight accuracy (see Figure S5), and sufficiently stability for the strength, closeness, EI, bridge strength and bridge EI centrality indices (see Figure S6) in the item‐level network (see Supporting Information Results and Figures S5 and S6).

**FIGURE 2:**
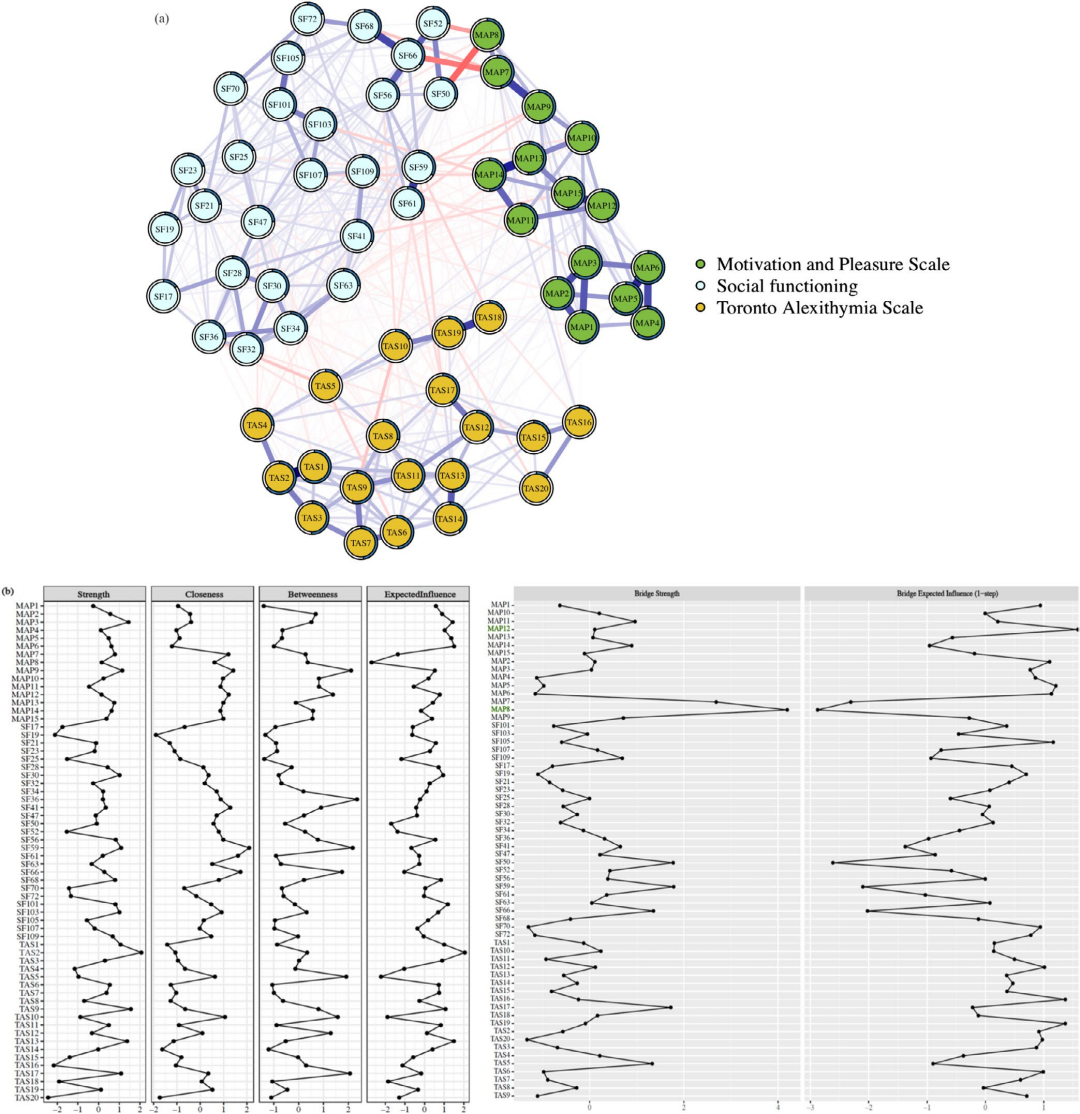
(a) Regularized partial correlation network of motivation and pleasure, alexithymia and social functioning at item‐level in college students (*n* = 2889). (b) The standardized centrality indices and bridge centrality indices of each node in the item‐level network. Each node in the network represents an item. Each edge represents the regularized partial correlation between two nodes controlled for all other nodes. Blue edges represent positive associations, whereas red edges represent negative associations. Thicker edges (positive and negative) signify stronger partial correlations. The pie chart surrounding each node represents node predictability. The value of each edge represents the value of the regularized partial correlation coefficients. MAP = The Motivation and Pleasure Scale—Self‐Report; SF = Social functioning measured by The Chinese version of the First‐Episode Social Functioning Scale; TAS = The Chinese version of 20‐item Toronto Alexithymia Scale.

The visualization of the network structure is presented in Figure 2a, the results concurred with the flow network. Generally, regarding the connections among the MAP, the TAS and social functioning, the MAP items was related to almost all FESFS subscales, in particular, the items belonging to the MAP “feelings about relationship” factor showed close connection with the items related to the FESFS “intimacy” (edge value = −0.253) and “family and friend” (edge value = −0.220) subscales. However, the TAS items showed weak negative connection with the FESFS subscales (edge values = −0.092—0.018). Regarding the centrality estimates (see Figure 2b), although Item 2 of the TAS showed the highest strength and EI centrality estimates, the items belonging to the MAP “feelings about relationship” and “motivation” factors showed the highest bridge strength and EI centrality indices.

### 3.5 Network Comparison Between Individuals With High SA and Low SA at Domain‐Level

**FIGURE 3:**
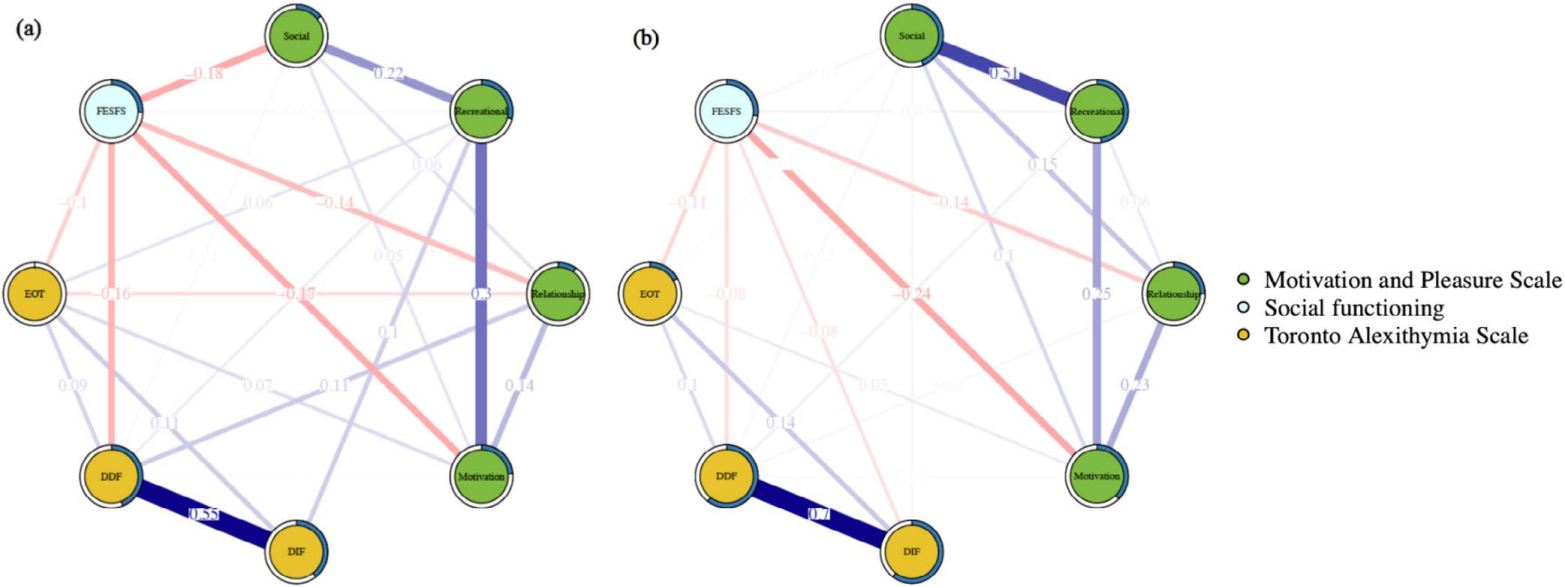
Regularized partial correlation networks for (a) high social anhedonia group (*n* = 246) and (b) low social anhedonia group (*n* = 1612). Each node represents a variable. Each edge represents the regularized partial correlation between two nodes controlled for all other nodes. Blue edges represent positive associations, whereas red edges represent negative associations. Thicker edges (positive and negative) signify stronger partial correlations. The pie chart surrounding each node represents node predictability. The value of each edge represents the strength of the regularized partial correlations. DDF = difficulty in describing feelings of TAS; DIF = difficulty in identifying feelings factor of TAS; EOT = externally oriented cognitive style thinking of TAS; FESFS = The Chinese version of the First‐Episode Social Functioning Scale; MAP‐SR = The Motivation and Pleasure Scale—Self‐Report; Motivation = motivation and effort in engaging in activities factor of MAP‐SR; Recreational = recreational/work pleasure factor of MAP‐SR; Relationship = feelings about relationship factor of MAP‐SR; Social = social pleasure factor of MAP‐SR; TAS = The Chinese version of 20‐item Toronto Alexithymia Scale.

Figure 3 shows the regularized networks estimated for the high SA and low SA subgroups. The two networks did not differ in global strength (Global strength difference = 0.350; global strength for high SA = 2.763, global strength for low SA = 3.113; *p* = 0.058); but these two networks differed in network structure (maximum edge‐weight difference = 0.287; *p* = 0.001), indicating that at least one edge was significantly different between these two networks. To further understand these differences, we checked the statistical difference at level of each single edge, by applying the Holm–Bonferroni corrections. The results did not suggest any statistical difference in 97% of the edges between the network of the high SA subgroup and that of the low SA subgroup. Both networks generally shared an overall similar edge structure. Only one edge was statistically different between the networks (*p* < 0.001), that is, the connection between the MAP “social pleasure” and “recreation/work pleasure” factors in high SA subgroup was significantly weaker than low SA subgroup (see Table S6).

## 4 Discussion

This study examined the interrelationship between the MAP, TAS, and FESFS in a large college student sample using network analysis and relative importance analysis. The estimated regularized flow network suggested that lower levels of motivation to engage in activities, rather than alexithymia, was more closely and negatively connected with social functioning. Moreover, the “item‐level” network further suggested that the MAP factors showed connections with all FESFS subscales, especially with FESFS “intimacy” and “family and friend” subscales. The centrality indices in our resultant networks suggested that, apart from social functioning, the MAP “motivation” and “feelings about relationship” factors played a key influential role. The relative importance analysis further suggested that the MAP “motivation” factor accounted for the highest proportion variance explained for social functioning.

Although the MAP factor has been consistently found to predict social functioning in clinical schizophrenia patients (Fervaha et al. 2015), how it interacts with schizotypal features to affect social functioning has seldom been studied. Using a large college student sample, we extended our earlier findings in clinical samples regarding the close connection between the MAP factors of negative symptoms and social functioning (Chang et al. 2020; Hu et al. 2022). In line with our previous results (Hu et al. 2022), we observed that social functioning was more strongly connected to the MAP “motivation” and “feelings about relationship” factors, and was weakly related to the TAS factors in the flow network. Furthermore, at the “item‐level” network, we found different connection patterns of the MAP and TAS items with various aspects of social functioning. Specifically, the MAP items was closely linked to almost all FESFS subscales, particularly the “intimacy” and “family and friend” domains, whereas the TAS items showed weak negative connected with the FESFS subscales. Besides, consistent with the previous results in clinical samples (Chang et al. 2020; Hu et al. 2022), the centrality analysis highlighted the central role of MAP factors with relatively high values of centrality indices (i.e., bridge strength, bridge EI), both in the flow network and the ‘item‐level’ network, supporting the important role of the MAP factor in social functioning across clinical and nonclinical samples. Taken together, the MAP factor may be a potential target for early intervention development.

Our NCT findings did not find any significant difference in global strength between subgroups with high and low SA, indicating that the absolute sum of network edge‐weights in high SA and low SA subgroups were not significantly different. However, the network structures between these two subgroups showed significant difference. When the corresponding edge‐weights of the two networks were further compared, we found the two networks having overall similar edge structure. Only one edge was statistically different in the network of the high SA subgroup, that is, the weaker connection between the MAP “social pleasure” and “recreational/work pleasure” factors, which is plausible that people with high SA were more inclined to gain pleasure from “nonsocial” activities (Llerena et al. 2012).

Our study has several limitations. First, we have not preregistered this study. Second, cross‐sectional design precluded inference of causality. Third, we did not perform strict sampling procedures and standardized assessments. Our sample had an unbalanced gender ratio, and males and females differed in age. Fourth, we only captured psychological phenomena using self‐report scales, which may be susceptible to social desirability and recall biases. Future research should employ laboratory‐based assessments, such as Effort‐Expenditure for Rewards Task (Treadway et al. 2009). Fifth, we did not account for the effects of past and current psychiatric treatments, and we did not measure participants' IQ. Finally, replicability of our network analysis findings remained unclear. Future network analysis research should include exploration and replication samples.

## 5 Conclusion

To conclude, the motivation factor is closely related to social functioning and could account for a larger proportion of variance of social functioning in a college student sample. Our findings suggested the important role of the motivation factor in social functioning, and highlighted the potential interaction between social functioning, MAP and alexithymia in a nonclinical sample.

## Conflicts of Interest

The authors declare no conflicts of interest.

## Supporting information


**Data S1.** Supporting Information.

**Data S2.** Supporting Information.
