# Supplementary material for: Motivation and Pleasure Domain Links to Social Function in College Students: A Network Analysis
Source: Psych J. 2025 Feb 24;14(4):534–44. doi: 10.1002/pchj.70001 (PMC12318595; doi:10.1002/pchj.70001)
Supplement: Supplementary file 1 — Data S1. Supporting Information. [file PCHJ-14-534-s002.docx]

**Supplementary Materials**

# S1. Supplementary Methods

**Exclusion criteria for Participants**

A total of 3901 college students were recruited. First, 272 participants who did not complete the school subscale of social functioning were excluded. Second, to screen for participants providing invalid responses, we incorporated the 13-item Chapman Infrequency Scale (CIS) (Chapman & Chapman, 1983) in our self-report measures. We excluded 469 participants from our sample because of they scored >3 on the CIS (Roivainen et al., 2016). Then, any participants with a self-account of current or past personal history of psychosis, or family history of psychosis would be excluded (n =271). Finally, 2889 eligible participants were retained in the final sample.

***Centrality estimates***

Four network centrality indices (i.e., strength, closesness, betweenness and expected influence) (Borsboom & Cramer, 2013; Robinaugh et al., 2016) were estimated to evaluate the importance of each node: (1) strength of a given node was calculated using the sum of the absolute edge weights of the particular node to all other nodes, and would reflect how strongly the particular node was directly connected to other nodes; (2) closeness was calculated as the inverse of the sum of distances of a given node from all other nodes in the network, indicating how easily the particular node could reach to all other nodes; (3) betweenness was calculated as how many times that a given node would lie on the shortest paths between two other nodes, indicating the importance of the particular node in linking all other nodes; (4) the expected influence (EI) was calculated using the sum of all the edge weights of a given node in the network, which the edge weight does not take absolute values. All these indices were transformed to standardized Z-scores.

Besides, we also calculate the bridge strength and bridge EI. Bridge strength is calculated as the sum of the absolute value of all edges that exist between a node A and all nodes that are not in the same domains/scales as node A. Bridge EI is calculated as the sum of edge values that exist between a node A and all nodes that are not in the same domains/scales as node A. Bridge EI accounted for the presence of negative edges (Jones et al., 2021).

The mean predictability of the overall network and the mean predictability of each node were also calculated with the R-package *mgm* (Haslbeck & Fried, 2017; Haslbeck & Waldorp, 2020), which is an absolute measure of interconnectedness of a given node, and indicates the variance of a given node that could be accounted for by the surrounding nodes.

***Network stability and accuracy***

Looking to estimate edge and centrality stability, R-package *bootnet* version 1.4.3 was used (Epskamp et al., 2018). Non-parametric bootstrap (resampling rows with replacement) was used to create 3000 samples to estimate edge weights stability. Case-dropping subset bootstrap samples (n = 3000) were used to estimate the stability of centrality indices. Correlation Stability (CS) coefficient for correlation values equal or above to *r* = 0.7 were used to measure stability of centrality indices (Epskamp et al., 2018). CS-coefficient indicates the percentage of our sample that can be dropped to maintain, with a 95% confidence interval, correlation values equal or above to *r* = 0.7 between our sample’s centrality indices and our bootstrapped samples' centrality indices.

**References:**

Borsboom, D., & Cramer, A. O. J. (2013). Network analysis: an integrative approach to the structure of psychopathology. *Annual Review of Clinical Psychology*, *9*, 91–121. https://doi.org/10.1146/annurev-clinpsy-050212-185608.

Chapman, L. J., & Chapman, J. P. (1983). Infrequence Scale (Unpublised test).

Epskamp, S., Borsboom, D., & Fried, E. I. (2018). Estimating psychological networks and their accuracy: A tutorial paper. *Behavior research methods*, *50*(1), 195–212. https://doi.org/10.3758/s13428-017-0862-1.

Haslbeck, J. M. B., & Fried, E. I. (2017). How predictable are symptoms in psychopathological networks? A reanalysis of 18 published datasets. *Psychological Medicine*, *47*(16), 2767–2776. https://doi.org/10.1017/S0033291717001258.

Haslbeck, J. M. B., & Waldorp, L. J. (2020). mgm: Estimating Time-Varying Mixed Graphical Models in High-Dimensional Data. *Journal of Statistical Software*, *93*(8), 1–46. https://doi.org/10.18637/jss.v093.i08

Jones, P. J., Ma, R., & McNally, R. J. (2021). Bridge Centrality: A Network Approach to Understanding Comorbidity. *Multivariate behavioral research*, *56*(2), 353–367. https://doi.org/10.1080/00273171.2019.1614898.

Robinaugh, D. J., Millner, A. J., & McNally, R. J. (2016). Identifying highly influential nodes in the complicated grief network. *Journal of Abnormal Psychology*, *125*(6), 747–757. https://doi.org/10.1037/abn0000181.

Roivainen, E., Veijola, J., & Miettunen, J. (2016). Careless responses in survey data and the validity of a screening instrument. *Nordic Psychology*, *68*(2), 114-123. https://doi.org/10.1080/19012276.2015.1071202.

S2. Supplementary Results

***Network accuracy and stability of the Flow network***

The results of the flow network accuracy and centrality indices stability were shown in the Supplementary Figure S1 – S2. As Figure S1 showed, the bootstrapped CIs were quite narrow, indicating reliable and accurate edge-weight estimates of the flow network. The CS-coefficients of edge accuracy was 0.75. The correlation stability (CS) coefficient for Strength, Closeness, Betweenness and Expected Influence (EI) were 0.750, 0.750, 0.517 and 0.750 respectively, all larger than 0.5 recommended by Epskamp et al. (2018), indicating that these four centrality indices have sufficient stability. The CS-coefficient for bridge strength, and EI were 0.750, 0.750 respectively. The predictability of nodes ranged from 17.50% (TAS-EOT) to 60.80% (TAS-DDF), with a mean predictability of 42.94% for the overall network.

***Network results of the Item-level network***

The results of the network accuracy and stability were estimated and described in Supplementary Figure S5 – S6. Regarding the results of the item-level network accuracy and centrality indices stability, the network showed relatively high accuracy for edge-weight estimates (Figure S5). The CS-coefficients of edge accuracy was 0.75. The item-level network had the sufficient stability for the Strength, Closeness centrality indices and EI (the CS-coefficients of 0.75 for Strength and 0.75 for EI, 0.517 for Closeness), not for the Betweenness centrality indices (CS-coefficients = 0.283). The bridge Strength (CS-coefficients = 0.75) and EI centrality (CS-coefficients = 0.75) indices also achieved adequate stability.

***Gender difference in network comparison***

The network comparison test for gender difference did not find any significant difference in terms of global strength (global strength difference = 0.153; male global strength = 3.228, female global strength = 3.382; *p* = .525), and network structure (maximum edge-weight difference = 0.089; *p* = .478).

# S3. Supplementary Tables

**Table S1.** The skewness, kurtosis and Cronbach’s 𝛼 for all the (sub)scales selected in the network (n = 2889)

|  | **Score range** | **Skewness** | **Kurtosis** | **Cronbach’s 𝜶** |
| --- | --- | --- | --- | --- |
| **MAP –** Social pleasure | 0 - 12 | 0.522 | -0.123 | 0.854 |
| **MAP –** Recreational or work pleasure | 0 - 12 | 0.330 | -0.403 | 0.882 |
| **MAP –** Feelings about relationship | 0 - 12 | 0.298 | -0.473 | 0.672 |
| **MAP –** Motivation | 0 - 23 | 0.010 | 0.032 | 0.818 |
| **TAS –** Difficulty in Identifying feelings (DIF) | 7 - 35 | 0.093 | -0.288 | 0.878 |
| **TAS –** Difficulty in Describing feelings (DDF) | 5 - 25 | 0.019 | -0.433 | 0.767 |
| **TAS –** Externally oriented cognitive style thinking (EOT) | 8 - 31 | -0.391 | 0.266 | 0.406 |
| **FESFS –** Interpersonal | 7 - 28 | 0.148 | 0.514 | 0.776 |
| **FESFS –** Family and friends | 7 - 20 | 0.075 | 0.010 | 0.701 |
| **FESFS -** Living skills | 4 - 16 | -0.089 | 0.271 | 0.574 |
| **FESFS –** Intimacy | 3 - 12 | -0.297 | 0.203 | 0.626 |
| **FESFS –** Balance | 3 - 12 | -0.136 | 0.708 | 0.485 |
| **FESFS -** School | 7 - 20 | 0.268 | 0.413 | 0.699 |
| **FESFS total score** | 47 – 108 | 0.431 | 0.553 | 0.874 |

*Abbreviations:* MAP-SR = The Motivation and Pleasure Scale - Self Report; TAS-20 = The Chinese version of 20-item Toronto Alexithymia Scale; FESFS = The Chinese version of the First-Episode Social Functioning Scale.

**Table S2.** Model fit results from Confirmatory Factor Analysis of the Measures used for the network

| **Models** | **AIC** | **BIC** | **𝜒^2^ (*df*)** | **CFI ^d^** | **TLI ^e^** | **RMSEA ^f^** | **SRMR** |
| --- | --- | --- | --- | --- | --- | --- | --- |
| 4-Factor of the MAP-SR ^a^ | 103941.649 | 104156.521 | 2099.907(84) | **0.904** | **0.881** | 0.091(0.088, 0.095) | 0.050 |
| 3-Factor of the TAS ^b^ | 148340.778 | 148597.431 | 4183.315(167) | **0.796** | **0.768** | 0.091(0.089, 0.094) | 0.122 |
| 6-factor of the FESFS ^c^ | 206024.030 | 206435.868 | 2689.960(309) | **0.866** | **0.848** | 0.052(0.050, 0.053) | 0.045 |

*Note:* ^a^ The 4-factor model of the MAP-SR includes social pleasure, recreational/work pleasure, feelings about relationship, and motivation factors.

^b^ The 3-factor model of the TAS includes Difficulty in Identifying feelings (DIF), Difficulty in Describing feelings (DDF), and Externally oriented cognitive style thinking (EOT) factors.

^c^ The 6-factor model of the FESFS organised into six subscales: Living skills, Interpersonal, Intimacy, Family and friends, School, and Balance.

^d^ CFI values ranged from 0 to 1, values greater than 0.90 indicate good fit.

^e^ TLI values greater than 0.90 indicating good fit. If the CFI and TLI are less than one, the CFI is always greater than the TLI.

^f^ The RMSEA is a parsimony index that evaluates the fit between the hypothesized model and the population covariance matrix. The information criteria are relative fit indices of model parsimony that take into account model complexity based on degrees of freedom.

*Abbreviations:* CFI = Comparative Fit Index; TLI = Tucker Lewis Index; RMSEA = Root Mean Square Error of Approximation; MAP-SR = The Motivation and Pleasure Scale - Self Report; TAS-20 = The Chinese version of 20-item Toronto Alexithymia Scale; FESFS = The Chinese version of the First-Episode Social Functioning Scale.

**Table S3.** Zero-order correlation matrix of all the variables selected for the flow network (n = 2889)

|  | **1** | **2** | **3** | **4** | **5** | **6** | **7** | **8** | **9** | **10** | **11** | **12** | **13** | **14** |
| --- | --- | --- | --- | --- | --- | --- | --- | --- | --- | --- | --- | --- | --- | --- |
| **1 MAP –** Social pleasure | 1.000 | -- | -- | -- | -- | -- | -- | -- | -- | -- | -- | -- | -- | -- |
| **2 MAP –** Recreational or work pleasure | 0.634 | 1.000 | -- | -- | -- | -- | -- | -- | -- | -- | -- | -- | -- | -- |
| **3 MAP –** Feelings about relationship | 0.471 | 0.404 | 1.000 | -- | -- | -- | -- | -- | -- | -- | -- | -- | -- | -- |
| **4 MAP –** Motivation | 0.487 | 0.528 | 0.483 | 1.000 | -- | -- | -- | -- | -- | -- | -- | -- | -- | -- |
| **5 TAS –** DIF | 0.304 | 0.266 | 0.240 | 0.225 | 1.000 | -- | -- | -- | -- | -- | -- | -- | -- | -- |
| **6 TAS –** DDF | 0.340 | 0.287 | 0.307 | 0.296 | 0.758 | 1.000 | -- | -- | -- | -- | -- | -- | -- | -- |
| **7 TAS –** EOT | 0.170 | 0.177 | 0.142 | 0.219 | 0.372 | 0.352 | 1.000 | -- | -- | -- | -- | -- | -- | -- |
| **8 FESFS –** Interpersonal | -0.344 | -0.294 | -0.314 | -0.423 | -0.304 | -0.378 | -0.267 | 1.000 | -- | -- | -- | -- | -- | -- |
| **9 FESFS –** Family and friends | -0.329 | -0.281 | -0.377 | -0.325 | -0.269 | -0.309 | -0.200 | 0.424 | 1.000 | -- | -- | -- | -- | -- |
| **10 FESFS –** Living skills | -0.247 | -0.214 | -0.179 | -0.245 | -0.248 | -0.250 | -0.222 | 0.419 | 0.406 | 1.000 | -- | -- | -- | -- |
| **11 FESFS –** Intimacy | -0.196 | -0.141 | -0.352 | -0.200 | -0.203 | -0.228 | -0.148 | 0.340 | 0.325 | 0.282 | 1.000 | -- | -- | -- |
| **12 FESFS –** Balance | -0.212 | -0.214 | -0.234 | -0.302 | -0.279 | -0.263 | -0.175 | 0.435 | 0.393 | 0.359 | 0.210 | 1.000 | -- | -- |
| **13 FESFS -** School | -0.309 | -0.291 | -0.300 | -0.410 | -0.293 | -0.286 | -0.201 | 0.486 | 0.552 | 0.406 | 0.290 | 0.501 | 1.000 | -- |
| **14 FESFS total score** | -0.400 | -0.349 | -0.420 | -0.466 | -0.380 | -0.417 | -0.295 | 0.797 | 0.740 | 0.667 | 0.563 | 0.647 | 0.765 | 1.000 |

*Note:* MAP-SR = The Motivation and Pleasure Scale - Self Report; TAS-20 = The Chinese version of 20-item Toronto Alexithymia Scale; TAS-DIF = Difficulty in identifying feelings; TAS-DDF = Difficulty in describing feelings; TAS-EOT = Externally oriented cognitive style of thinking; FESFS = The Chinese version of the First-Episode Social Functioning Scale.

**Table S4.** Centrality, expected influence(EI), bridge centrality indices and predictability of each node in the Flow Network (n = 2889)

|  | **Strength** | **Closeness** | **Betweenness** | **EI** | **Bridge strength** | **Bridge EI** | **Predictability** |
| --- | --- | --- | --- | --- | --- | --- | --- |
| **MAP** – Social pleasure | 0.497 | -0.484 | -0.636 | 0.632 | -0.487 | 0.508 | 0.484 |
| **MAP –** Recreational or work pleasure | -0.078 | -0.121 | -0.071 | **0.756** | -1.068 | **0.680** | 0.468 |
| **MAP –** Feelings about relationship | -0.597 | 0.265 | -0.919 | -0.114 | -0.166 | 0.054 | 0.338 |
| **MAP –** Motivation | 0.679 | 1.280 | 0.778 | 0.039 | 0.344 | -0.114 | 0.420 |
| **TAS –** DIF | 0.847 | -0.964 | -0.636 | 0.674 | -0.489 | 0.345 | 0.585 |
| **TAS –** DDF | **0.940** | -0.574 | 0.495 | 0.683 | -0.057 | 0.653 | **0.608** |
| **TAS –** EOT | -2.069 | -1.044 | -0.919 | -0.460 | -0.338 | 0.251 | 0.175 |
| **FESFS total score** | -0.219 | **1.641** | **1.909** | -2.210 | **2.262** | -2.378 | 0.357 |

*Note:* MAP-SR = The Motivation and Pleasure Scale - Self Report; TAS-20 = The Chinese version of 20-item Toronto Alexithymia Scale; TAS-DIF = Difficulty in identifying feelings; TAS-DDF = Difficulty in describing feelings; TAS-EOT = Externally oriented cognitive style of thinking; FESFS = The Chinese version of the First-Episode Social Functioning Scale.

**Table S5.** Bootstrap confidence intervals (CIs) of difference between relative contributions of predictors to the social functioning in college students sample (n = 2889)

| **Pairs of predictors** | **Differences of Contributions** | **90% CIs** |
| --- | --- | --- |
| DIF – DDF | -0.011 | (-0.022, -0.000) |
| **DIF - EOT** | **0.014^*^** | (0.002, 0.026) |
| DIF – Social pleasure | -0.001 | (-0.014, 0.012) |
| **DIF – Recreational or work pleasure** | **0.016^*^** | (0.005, 0.027) |
| **DIF – Feelings about relationship** | **-0.019^*^** | (-0.033, -0.005) |
| **DIF – Motivation** | **-0.044^*^** | (-0.061, -0.026) |
| **DDF - EOT** | **0.025^*^** | (0.013, 0.039) |
| DDF – Social pleasure | 0.011 | (-0.002, 0.024) |
| **DDF – Recreational or work pleasure** | **0.027^*^** | (0.017, 0.040) |
| DDF – Feelings about relationship | -0.008 | (-0.022, 0.007) |
| **DDF – Motivation** | **-0.032^*^** | (-0.051, -0.014) |
| **EOT – Social pleasure** | **-0.015^*^** | (-0.027, -0.003) |
| EOT – Recreational or work pleasure | 0.002 | (-0.008, 0.012) |
| **EOT – Feelings about relationship** | **-0.033^*^** | (-0.047, -0.019) |
| **EOT – Motivation** | **-0.058^*^** | (-0.075, -0.041) |
| **Social pleasure – Recreational or work pleasure** | **0.017^*^** | (0.009, 0.025) |
| **Social pleasure – Feelings about relationship** | **-0.019^*^** | (-0.033, -0.005) |
| **Social pleasure – Motivation** | **-0.043^*^** | (-0.059, -0.027) |
| **Recreational or work pleasure – Feelings about relationship** | **-0.035^*^** | (-0.047, -0.023) |
| **Recreational or work pleasure – Motivation** | **-0.060^*^** | (-0.075, -0.045) |
| **Feelings about relationship – Motivation** | **-0.024^*^** | (-0.043, -0.007) |

*Note:* The CIs for differences show which differences in contributions are considered statistically significant (in an exploratory sense). * indicates that those contributions for which the confidence interval does not contain the equality case. MAP-SR = The Motivation and Pleasure Scale - Self Report, comprising 4 factors: social pleasure, recreational or work pleasure, feelings about relationship, and motivation; TAS-20 = The Chinese version of 20-item Toronto Alexithymia Scale; DIF = Difficulty in identifying feelings of TAS-20; DDF = Difficulty in describing feelings of TAS-20; EOT = Externally oriented cognitive style of thinking of TAS-20.

**Table S6.** Network comparison between individuals with high SA (n = 246) and low SA (n = 1612)

|  | **Weights in high SA** | **Weights in low SA** | ***p* ^a^** | ***p* ^b^** |
| --- | --- | --- | --- | --- |
| DDF - DIF | 0.549 | 0.702 | .004 | .081 |
| DDF - EOT | 0.088 | 0.096 | .889 | 1 |
| DDF - FESFS | -0.158 | -0.081 | .198 | 1 |
| DDF - Social pleasure | 0.010 | 0.019 | .879 | 1 |
| DDF - Recreational or work pleasure | 0.036 | 0.037 | .984 | 1 |
| DDF - Feelings about relationship | 0.109 | 0.021 | .027 | .576 |
| DDF – Motivation | 0.003 | 0.007 | .910 | 1 |
| DIF – EOT | 0.107 | 0.144 | .604 | 1 |
| DIF – FESFS | 0 | -0.080 | .165 | 1 |
| DIF - Social pleasure | 0 | 0.021 | .475 | 1 |
| DIF – Recreational or work pleasure | 0.102 | 0 | .011 | .375 |
| DIF - Feelings about relationship | 0 | 0 | 1 | 1 |
| DIF – Motivation | 0 | 0 | 1 | 1 |
| EOT - FESFS | -0.098 | -0.108 | .875 | 1 |
| EOT - Social pleasure | 0 | 0.009 | .358 | 1 |
| EOT - Recreational or work pleasure | 0.057 | 0 | .103 | 1 |
| EOT - Feelings about relationship | -0.077 | 0 | .064 | 1 |
| EOT – Motivation | 0.072 | 0.048 | .700 | 1 |
| FESFS - Social pleasure | -0.183 | -0.027 | .003 | .081 |
| FESFS - Recreational or work pleasure | -0.016 | -0.034 | .760 | 1 |
| FESFS - Feelings about relationship | -0.138 | -0.140 | .970 | 1 |
| FESFS – Motivation | -0.173 | -0.235 | .355 | 1 |
| **Social pleasure – Recreational or work pleasure** | **0.225** | **0.512** | **0** | **< .001** |
| Social pleasure - Feelings about relationship | 0.059 | 0.154 | .171 | 1 |
| Social pleasure – Motivation | 0.055 | 0.103 | .436 | 1 |
| Recreational or work pleasure - Feelings about relationship | 0 | 0.058 | .387 | 1 |
| Recreational or work pleasure – Motivation | 0.305 | 0.251 | .379 | 1 |
| Feelings about relationship – Motivation | 0.144 | 0.227 | .202 | 1 |

*Note:* ^a^ *p* value without controlling for multiple edges; ^b^ *p* value after applying the Holm-Bonferroni correction.

MAP-SR = The Motivation and Pleasure Scale - Self Report, comprising 4 factors: social pleasure, recreational or work pleasure, feelings about relationship, and motivation; TAS-20 = The Chinese version of 20-item Toronto Alexithymia Scale; DIF = Difficulty in identifying feelings; DDF = Difficulty in describing feelings; EOT = Externally oriented cognitive style of thinking.

# S4. Supplementary Figures


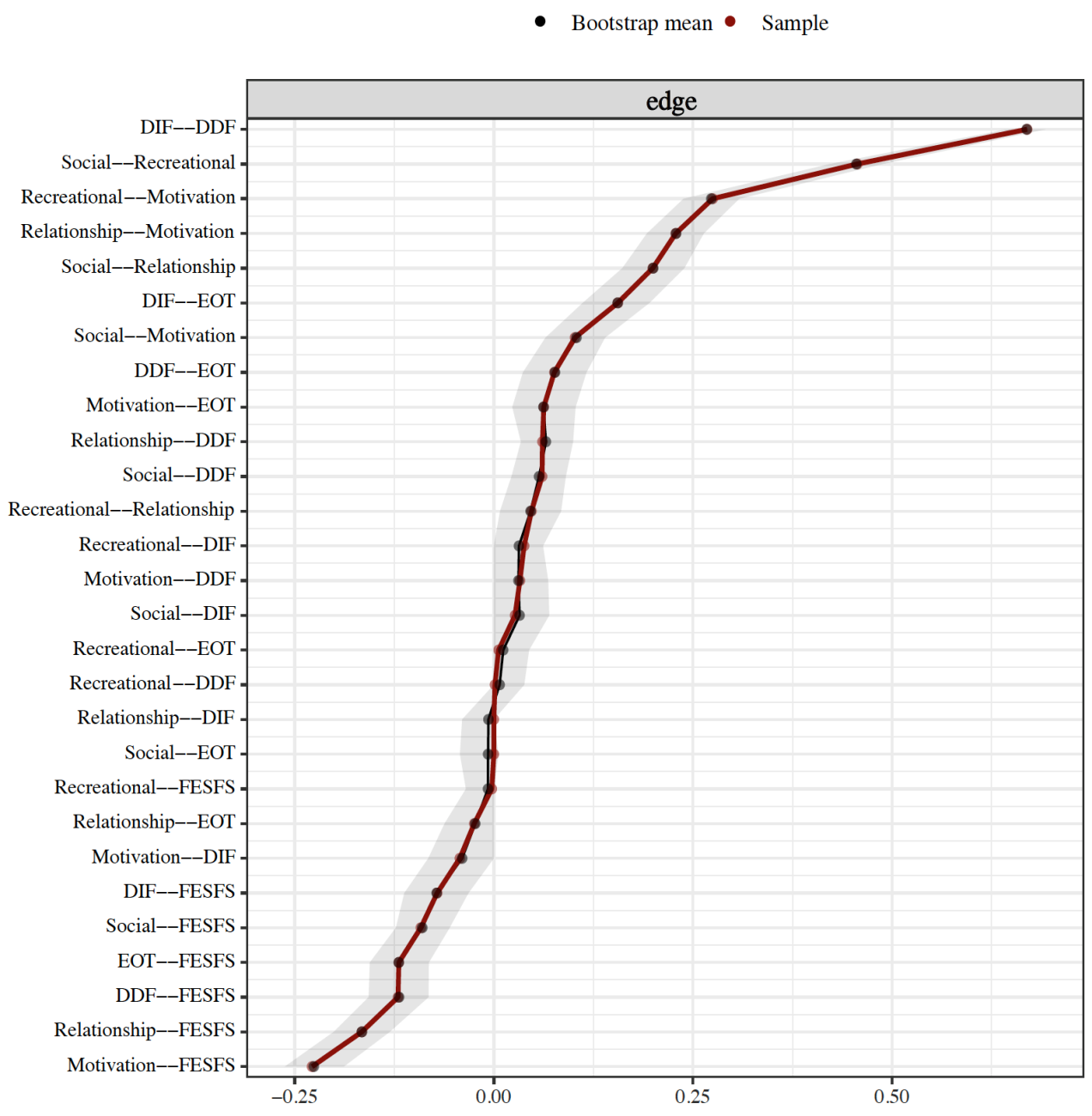


**Figure S1.** Bootstrapped 95% confidence intervals of edge weights of the flow network.

*Note:* Y-axis presents the labels of all edges in the network, ordered from the highest edge (top) to the lowest edge (bottom). Red dots are the edge weights of the estimated network; the grey area indicates the 95% CI around the edge weights.

**Figure S2.** The stability of centrality indices assessed by case-dropping subset bootstrap procedure in the flow network.

*Note:* Average correlations (Y-axis) between centrality indices of the original network for the whole sample and those estimated from networks for subsamples with participants dropped (X-axis, percentages of sampled participants). Lines indicate values of average correlations and areas indicate range from the 2.5th quantile to the 97.5th quantile.


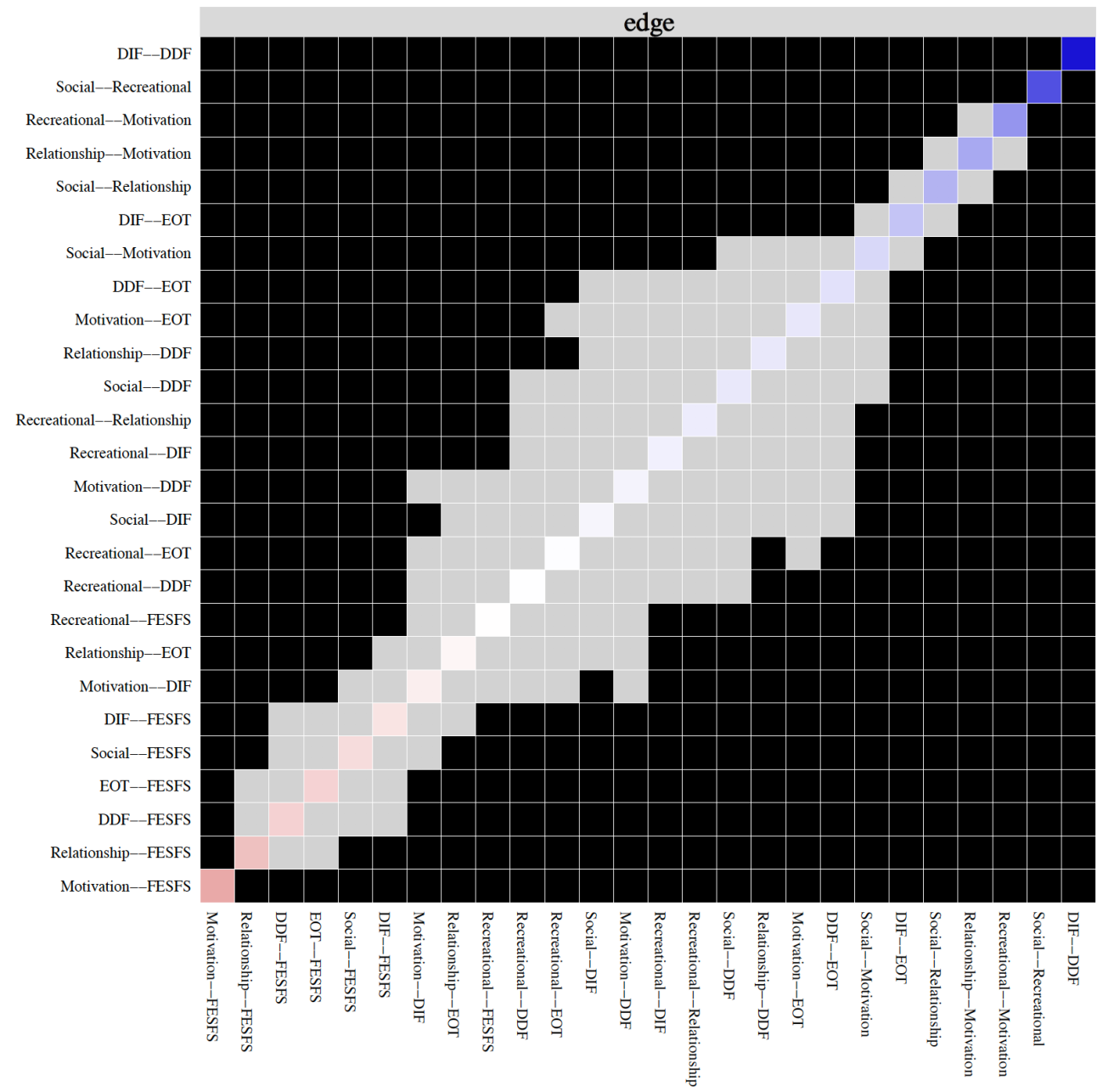


**Figure S3.** Bootstrapped difference test between edge-weights in the Flow Network.

*Note:* Grey boxes indicate edges that do not differ significantly from one-another; black boxes represent edges that do differ significantly from one-another.


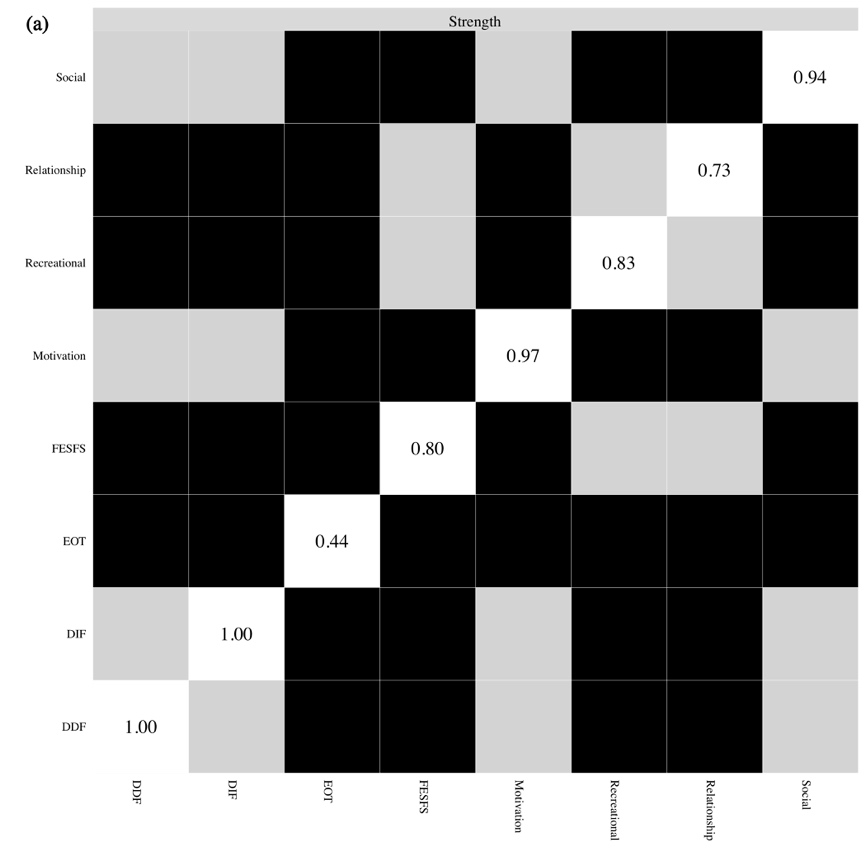

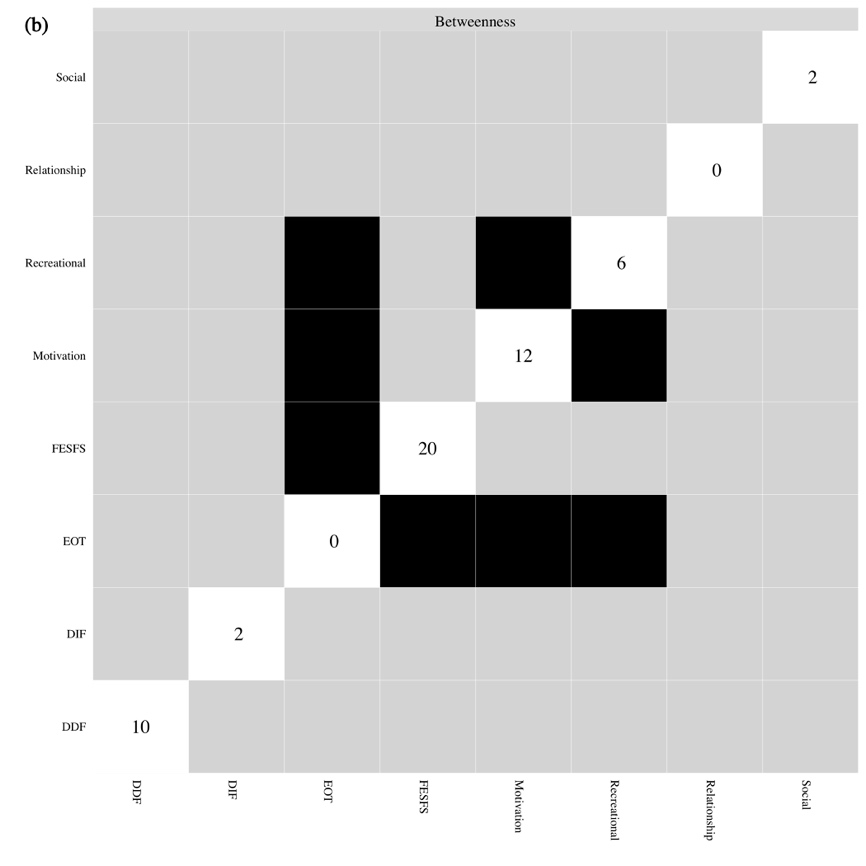

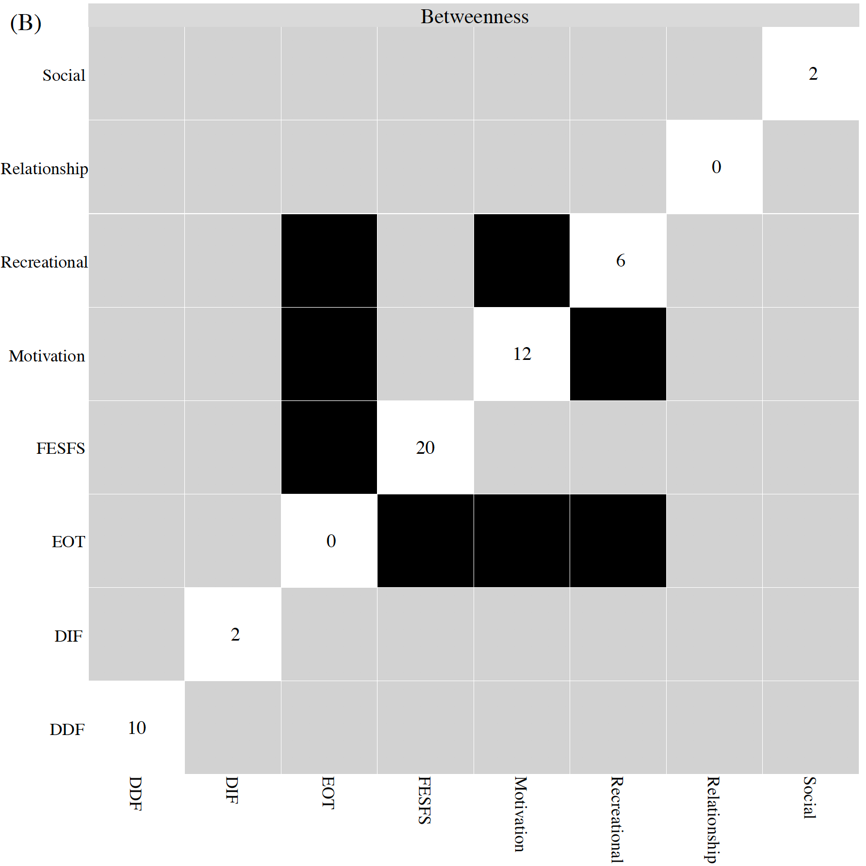


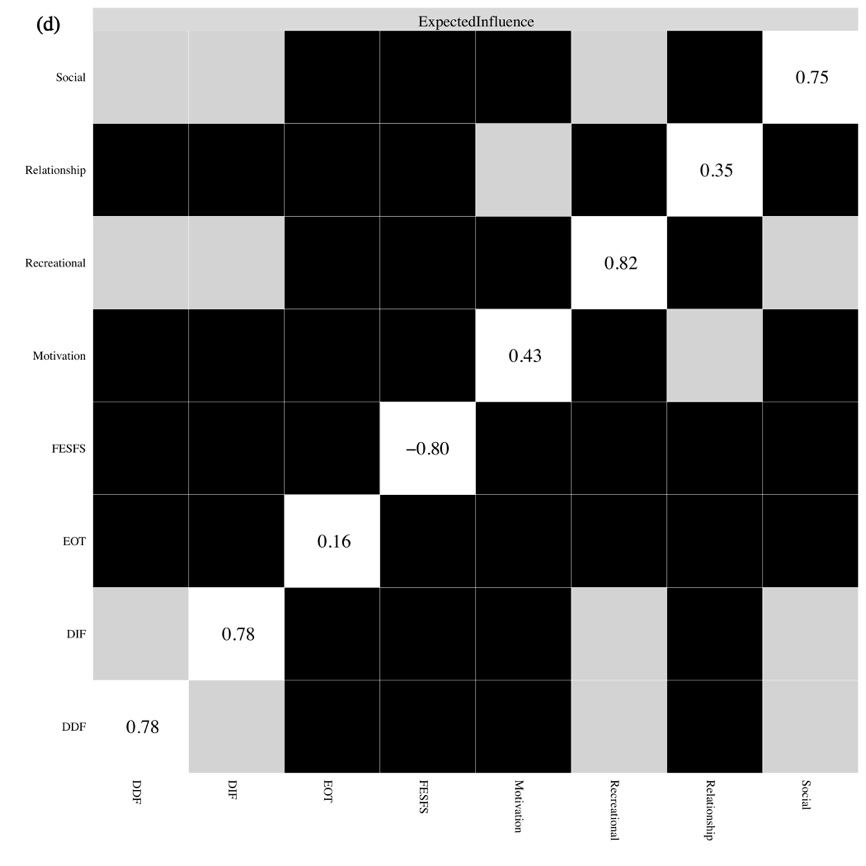

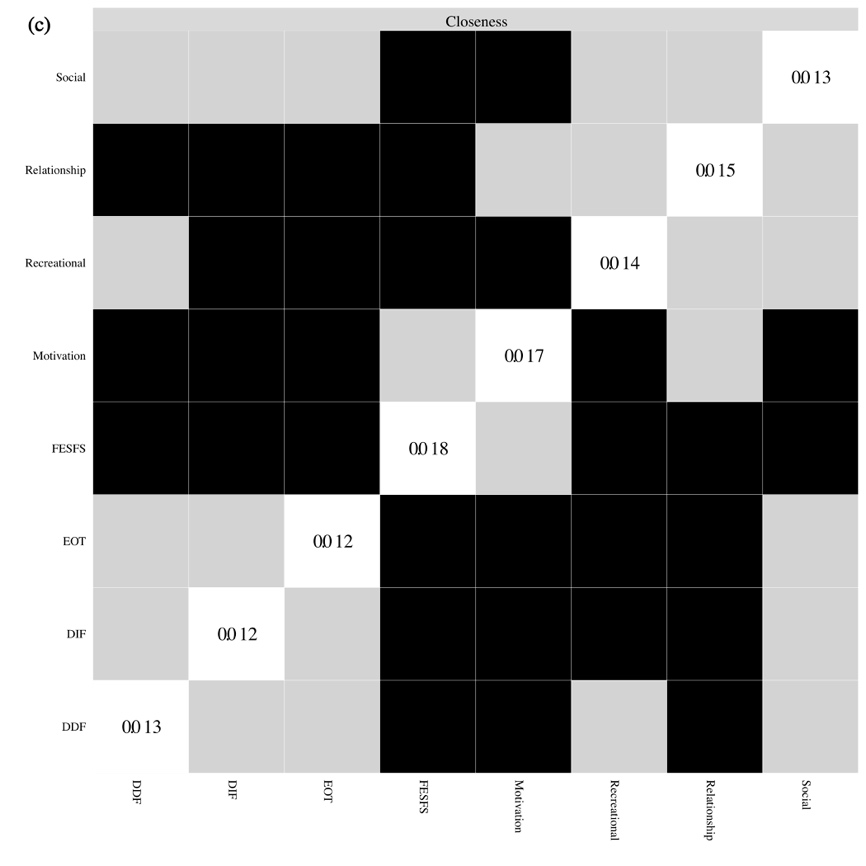


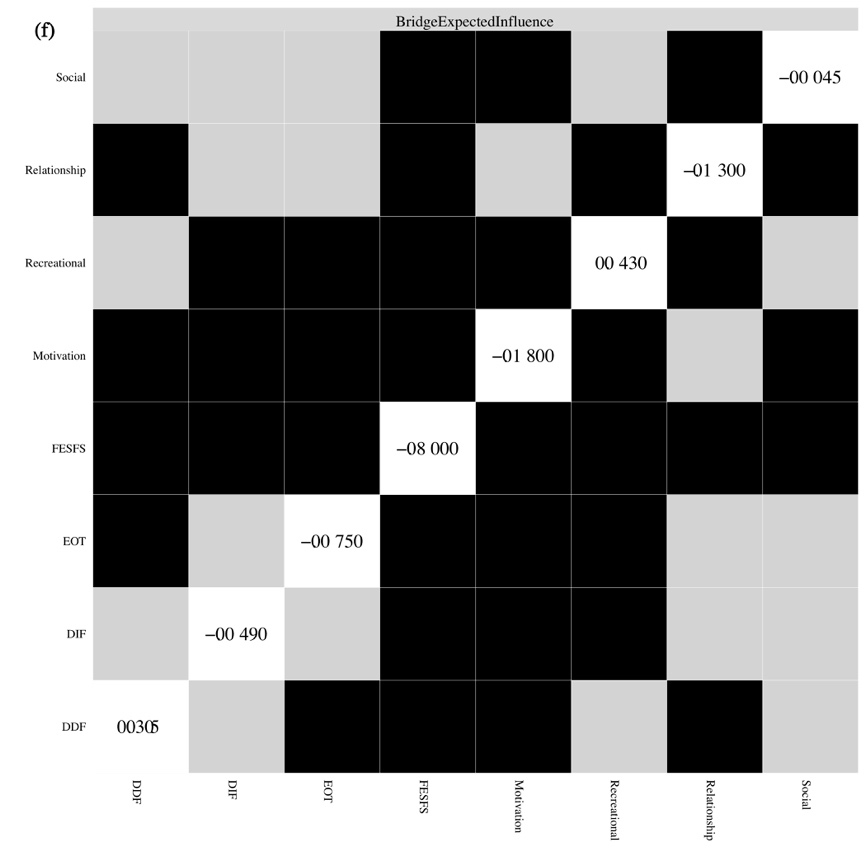

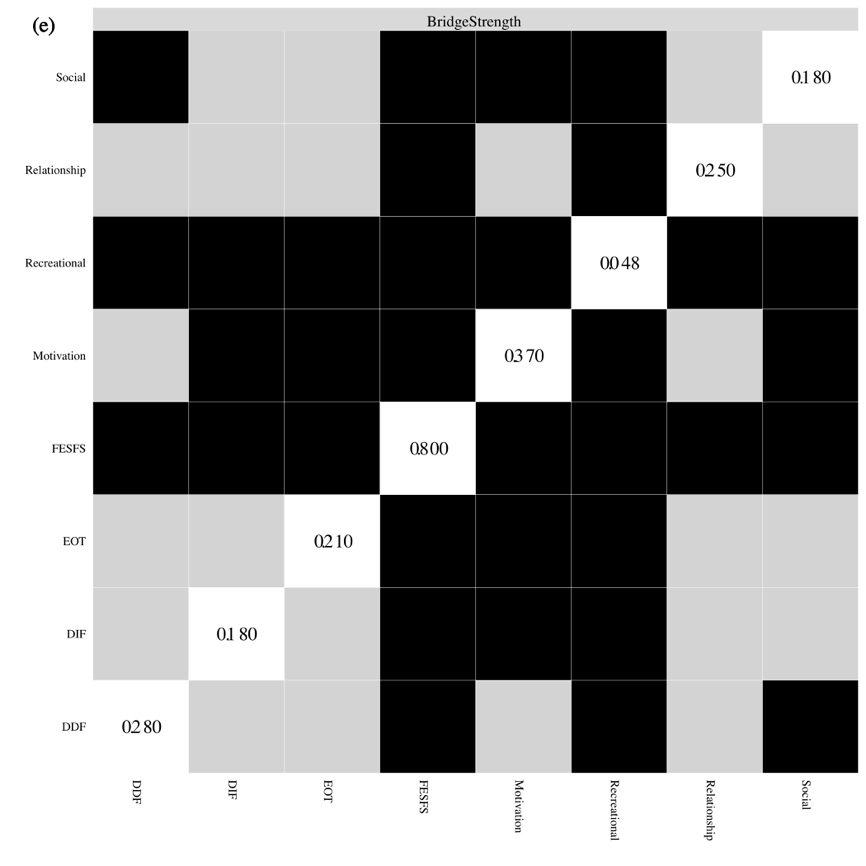


**Figure S4.** Bootstrapped difference tests between node centrality indices (a) strength, (b) betweenness, (c) closeness, (d) expected influence and (e) bridge strength, and (f) bridge expected influence in the flow network

*Note:* Grey boxes indicate nodes that do not differ significantly from one-another and black boxes represent nodes that do differ significantly from one-another. White boxes in the centrality plot show the value of estimated node strength, betweenness, closeness and expected influence.

**Figure S5.** Bootstrapped 95% confidence intervals of edge weights of the item-level network.

*Note:* Y-axis represents the edges in the network, ordered from the highest edge-weight (top) to the lowest edge-weight (bottom). The y-axis labels have been removed to avoid cluttering. Red dots are the edge weights of the estimated network; the grey area indicates the 95% confidence intervals around the edge weights.

**Figure S6.** The stability of centrality indices assessed by case-dropping subset bootstrap procedure in the item-level network.

*Note:* Average correlations (Y-axis) between centrality indices of the original network for the whole sample and those estimated from networks for subsamples with participants dropped (X-axis, percentages of sampled participants). Lines indicate values of average correlations and areas indicate range from the 2.5th quantile to the 97.5th quantile.
