# Supplementary material for: Motivation and Pleasure Domain Links to Social Function in College Students: A Network Analysis
Source: Psych J. 2025 Feb 24;14(4):534–44. doi: 10.1002/pchj.70001 (PMC12318595; doi:10.1002/pchj.70001)
Supplement: Supplementary file 2 — Data S2. Supporting Information. [file PCHJ-14-534-s001.docx]

**#R Script --** **Network estimates, visualization, centrality estimates, network comparison and relative importance analysis**

###################

**#**Import libraries (Please make sure you have installed these packages first) ** #**

###################

library(dplyr)

library(tibble)

library(ggplot2)

library(qgraph)

library(bootnet)

library(networktools)

library(igraph)

library(NetworkComparisonTest)

library(huge)

library(extrafont)

library(mgm)

library(relaimpo)

########################################################################

**# Network estimation, visualization, centrality estimates**

########################################################################

**# 1 A flow network estimation to elucidate the effects of MAP and TAS factors on social functioning (n = 2889) #**

########################################################################

# read data - standardized Z score of MAP factors, TAS factors and FESFS

a <- read.csv ('source path file.csv', header = TRUE)

# rename each variable included in the flow network

names(a) <- c("Social","Recreational","Relationship","Motivation","DIF","DDF","EOT","FESFS")

**# By using partial correlation and then conducting EBICglasso method to estimate network #**

mynetwork <- estimateNetwork(a,default = 'EBICglasso',tuning = 0.5 )

Weights <- getWmat(mynetwork)

write.csv(Weights,file = 'Flow_network_edge_weights_value.CSV',row.names = TRUE)

**# Compute the correlation matrix of the flow network shown in Table S3 #**

corNet <- cor_auto (a, missing = "pairwise")

**# Predictability of each node calculated in the flow network shown in Table S4 #**

dl <- list (data = a)

set.seed(1)

fit <- mgm(data = dl$data, type = c(rep("g",8)), level = c(rep("1",8)), lambdaSel='EBIC', lambdaGam = 0.5, ruleReg = 'AND', k = 2, binarySign = TRUE, overparameterize = FALSE)

**# For continuous variables, we specify explained variance ("R2") as predictability measure for** **each node #**

pred <- predict(object = fit, data = dl$data,errorCon = c("RMSE","R2"),errorCat = c("CC","nCC"))

R2 <-pred$errors

write.csv (R2, file = 'Predictability_in_Flow_network.CSV', row.names = TRUE)

mean (pred$errors [,3]) # mean predictability of the network

**# Network Visualization - the flow network in the entire sample (n = 2889) shown in Figure1 #**

# Grouping nodes: four nodes belonging to MAP factor, three nodes belonging to TAS

col <- c("Motivation and Pleasure Scale","Motivation and Pleasure Scale","Motivation and Pleasure Scale","Motivation and Pleasure Scale","Toronto Alexithymia Scale","Toronto Alexithymia Scale","Toronto Alexithymia Scale","Social functioning")

**# Plot flow network shown in Figure 1(a) of the main text #**

myplot <- plot(mynetwork, layout = 'spring', groups = col, color = c("#7FBC41","#DFFFFFFF", "#E8C32EFF"), theme = "Borkulo", pie = pred$errors$R2, pieColor=rep('#377EB8',8), border.color = "black", GLratio = 2.0,legend.cex = 0.28, labels = colnames(a), vsize = 7,label.cex = 1,label.font = 16,label.scale.equal = TRUE, label.scale = TRUE, edge.labels = TRUE, edge.label.cex=0.8, layoutScale = c(0.88,0.75), layoutOffset = c(-0.23,0))

pdf("Flow_Network.pdf", width = 7,height = 5.6, family='Times')

flow <- flow(myplot,"FESFS", horizontal = TRUE, theme = "Borkulo", layoutScale = c(0.5,0.78), vsize = 7,layoutOffset = c(-0.5,0), legend.cex = 0.3,curveShape =-1,curvePivot = 0.25,curvePivotShape=2.0)

dev.off()

**# Node centrality estimation in the flow network - Create centrality plot shown in Figure 1(b) #**

pdf("Centrality_with_flow_network.pdf",width = 5.8,height = 5.6, family='Times')

c <- centralityPlot(myplot, include = c("Strength","Closeness","Betweenness","ExpectedInfluence"), scale = "z-scores", orderBy = "default", decreasing = TRUE)

dev.off()

**# bridge strength and bridge expected influence estimation of the nodes in the flow network**

bridge_centrality_one_score <- bridge(Weights, communities=col)

plot(bridge_centrality_one_score, include=c("Bridge Strength", "Bridge Expected Influence (1-step)"), zscore=TRUE, order = "alphabetical")

**# The standardized Z-scores of node centrality indices in the flow network shown in the Table S4 #**

d <- centralityTable(mynetwork)

write.csv (d, file = 'CentralityTable.CSV', row.names = FALSE, scale = "z-scores")

bridgeStrength_raw_score <- bridge_centrality_one_score$`Bridge Strength`

bridgeStrength_Zscore <- (bridgeStrength_raw_score-mean(bridgeStrength_raw_score))/sd(bridgeStrength_raw_score)

bridgeEI_raw_score <- bridge_centrality_one_score$`Bridge Expected Influence (1-step)`

bridgeEI_Zscore <- (bridgeEI_raw_score-mean(bridgeEI_raw_score))/sd(bridgeEI_raw_score)

bridge_centrality_one_score_Table <- cbind(bridgeStrength_Zscore, bridgeEI_Zscore)

**# Network stability and accuracy - Generate 95% confidence intervals (CIs) for edge-weights to examine their stability, by default, non-parametric bootstrap is used #**

e <- bootnet (mynetwork, nBoots = 3000, statistics = "all", communities = col, nCores=1)

save (e, file ="edgeStability1.Rdata")

**# Plot the edge-weights accuracy results shown in the Figure S1 #**

pdf("edgeStability.pdf", family = "Times")

plot(e, labels = TRUE, order = "sample")

dev.off()

**# Network stability and accuracy Quantify accuracy by generating CS coefficient, by default a correlation of 0.7 between the bootstrap sample and the original sample was tested #**

f <- bootnet(mynetwork, nBoots = 3000, type = "case", statistics =c("edge","strength","closeness","betweenness","expectedInfluence","bridgeStrength","bridgeExpectedInfluence"), communities = col, nCores = 1)

save (f,file ="CentralStability1.Rdata")

corStability(f, statistics = "all")

**# Plot the network stability results shown in the Figure S2**

pdf("centrStability.pdf",family = "Times")

plot(f, statistics =c("strength","closeness","betweenness","expectedInfluence","bridgeStrength","bridgeExpectedInfluence"), rank = TRUE)

dev.off()

**# Plot bootstrapped differences of edge-weights shown in Figure S3 #**

pdf("Flow_network_edgedifference_test.pdf", family = "Times")

plot(e, "edge", plot = "difference", onlyNonZero = TRUE, order = "sample")

dev.off()

**# Plot significant differences of node Centrality indices (including Strength, closeness, betweenness, expected influence, bridge strength and bridge EI) shown in Figure S4 #**

pdf("Total_sample_final/Centrality_indices_difference.pdf", family='Times')

plot(e,"strength", labels = TRUE)

plot(e,"betweenness", labels = TRUE)

plot(e,"closeness", labels = TRUE)

plot(e,"expectedInfluence", labels = TRUE)

plot(e, "bridgeStrength", plot = "difference")

plot(e, "bridgeExpectedInfluence", plot = "difference")

dev.off()

#####################################################################

**# 2 Relative importance analysis in the entire sample (n = 2889) (shown in Table 2) #**

#####################################################################

**# Linear regression coefficients in the entire sample shown in the Table 2 #**

regression <-lm(formula = FESFS~DIF+DDF+EOT+Social+Recreational+Relationship+Motivation, data = a)

coefficients <- summary(regression)

write.csv (coefficients$coefficients, "coefficients.csv")

**# Relative importance of each variable (explained R^2^) in the entire sample (n = 2889) #**

RI_for_FESFS <-boot.relimp(FESFS~DIF+DDF+EOT+Social+Recreational+Relationship+Motivation, a, type = "lmg", diff = TRUE, rela = FALSE)

diff <- booteval.relimp(RI_for_FESFS, typesel = c("lmg"), level = 0.9, bty = "perc",norank = TRUE)

**# The quantitative contribution of each variable (explained R^2^) shown in the Table 2#**

write.csv(diff@lmg,"lmg.csv")

**# Pairwise differences in relative importance of variables, bootstrap confidence intervals of differences shown in Table S5 #**

write.csv(diff@lmg.diff, "lmg_diff.csv")

write.csv(diff@lmg.diff.lower, "diff_lower.csv")

write.csv(diff@lmg.diff.upper, "diff_upper.csv")

###############################################################################

**# 3 Regularised partial correlation network at item-level #**

###############################################################################

**# read data #**

b <- read.csv('Network_analysis_in_nonclinical_2889_item_level.csv',header = TRUE)

**#Network estimation**

network_item_level <- estimateNetwork(b,default = 'EBICglasso',tuning = 0.5)

#Network edge-weights

Weights_item_level <- getWmat(network_item_level)

write.csv(Weights_item_level,file = 'Weights_item_level.CSV', row.names = TRUE)

**#predictability**

dl_item <- list(data = b)

set.seed(1)

fit_item <- mgm(data = dl_item$data, type = c(rep("g",62)), evel = c(rep("1",62)), lambdaSel = "EBIC", lambdaGam = 0.5, ruleReg = 'AND', k = 2, binarySign = TRUE, overparameterize = FALSE)

**# For continuous variables, we specify explained variance ("R2") as predictability measure.**

pred_item <- predict(fit_item, dl_item$data)

R2_item_level <-pred_item$errors

mean(pred_item$errors[,3]) # mean predictability

**# Network Visualization – the item-level network in the entire sample (n = 2889) shown in Figure 2(a) #**

library(extrafont)

col_item_level <- c(rep("Motivation and Pleasure Scale", 15), rep("Toronto Alexithymia Scale", 20), rep("Social functioning", 27))

pdf("Network_analysis_item_level.pdf",width = 11,height = 8.5, family='Times')

plot_item_level <- plot(network_item_level, layout = 'spring',groups = col_item_level, theme = "Borkulo",color = c("#E8C32EFF","#7FBC41","#DFFFFFFF"), pie = pred_item$errors$R2,pieColor=rep('#377EB8',62), border.color = "black", legend.cex = 0.3, vsize = 5, label.cex = 1.1, label.scale.equal = TRUE,label.scale = TRUE,edge.labels = FALSE,edge.label.cex=0.5)

dev.off()

**# Centrality estimates - Create centrality plot shown in Figure 2(b) #**

pdf("centrality.pdf",width = 7,height = 8.5, family='Times')

c_item_level <- centralityPlot(plot_item_level, include = c("Strength","Closeness","Betweenness","ExpectedInfluence"),scale = "z-scores", decreasing = TRUE)

dev.off()

#Bridge centrality estimation

bridge_centrality_item_level <- bridge(Weights_item_level, communities=col_item_level)

pdf("bridge_centrality.pdf",width = 7,height = 8.5, family='Times')

plot(bridge_centrality_item_level,include=c("Bridge Strength","Bridge Expected Influence (1-step)"),zscore=TRUE,order = "alphabetical")

dev.off()

**# Network stability and accuracy - Generate 95%CI for edge-weights to examine their accuracy, by default, non-parametric bootstrap is used (As Figure S5 is shown) #**

e2 <- bootnet(network_item_level,nBoots = 3000,statistics = "all",communities = col_item_level)

pdf("edgeStability_item_level.pdf",width = 7,height = 10, family='Times')

plot(e2,labels = FALSE, order = "sample")

dev.off()

**# Network stability and accuracy - Quantify stability by generating CS coefficient, by default a correlation of 0.7 between bootstrap sample and original sample was tested (Figure S6 is shown)**

f2 <- bootnet(network_item_level, nBoots = 3000, type = "case",statistics = "all",communities = col_item_level)

corStability(f2,statistics = "all")

pdf("centrStability_item_level.pdf",width = 7,height = 6, family='Times')

plot(f2,statistics =c("strength","closeness","betweenness","expectedInfluence","bridgeStrength","bridgeExpectedInfluence"),rank = TRUE)

dev.off()

###############################################################################

**# 4 Network comparison between Subgroups the subgroup with high levels of Social Anhedonia (SA) and the subgroup with low levels of SA #**

###############################################################################

# read data #

highSA <- read.csv('source path/highSA.csv', header = TRUE)

lowSA <- read.csv('source path/lowSA.csv', header = TRUE)

# rename each variable in the dataframe #

names(highSA) <- c("Social", "Recreational", "Relationship", "Motivation", "DIF", "DDF", "EOT", "FESFS")

names(lowSA) <- c("Social", "Recreational", "Relationship", "Motivation", "DIF", "DDF", "EOT", "FESFS")

# Estimate the network of each group #

network_highSA <- estimateNetwork(highSA, default = 'EBICglasso', tuning = 0.5)

network_lowSA <- estimateNetwork(lowSA, default = 'EBICglasso', tuning = 0.5)

# Edge weights of networks of high SA and low SA (Shown in Table S6) #

weight_highSA <- getWmat(network_highSA)

weight_lowSA <- getWmat(network_lowSA)

**# Network_comparison_test_without_controlling_for_multiple_edges** (p value Shown in Table S6) **#**

myNCT <- NCT(network_highSA, network_lowSA, it=1000, weighted = TRUE, test.edges = TRUE, edges = "ALL", p.adjust.methods = "none", test.centrality = TRUE)

summary(myNCT)

p_value <- myNCT[["einv.pvals"]]

**# Network_comparison_test_applying_Holm-Bonferroni correction** (adjusted p value Shown in Table S6) **#**

myNCT1 <- NCT(network_highSA, network_lowSA, it=1000, weighted = TRUE, test.edges = TRUE, edges = "ALL", p.adjust.methods = "holm", test.centrality = TRUE)

summary(myNCT1)

p_value_adjusted <- myNCT1[["einv.pvals"]]

**# Network visualization of these two subgroup (highSA and lowSA) shown in Figure 3 #**

# Calculate the predictability of each node in the network of highSA subgroup #

dl_highSA <- list(data = highSA)

set.seed(1)

fit_highSA <- mgm(data = dl_highSA$data, type = c(rep("g",8)), level = c(rep("1",8)), lambdaSel='EBIC', lambdaGam = 0.5, ruleReg = 'AND', k = 2, binarySign = TRUE, overparameterize = FALSE)

pred_highSA <- predict(object = fit_highSA, data = dl_highSA$data, errorCon = c("RMSE","R2"), errorCat = c("CC","nCC"))

R2_highSA <-pred_highSA$errors

# Calculate the predictability of each node in the network of lowSA subgroup #

dl_lowSA <- list(data = lowSA)

set.seed(1)

fit_lowSA <- mgm(data = dl_lowSA$data, type = c(rep("g",8)), level = c(rep("1",8)), lambdaSel='EBIC', lambdaGam = 0.5, ruleReg = 'AND', k = 2, binarySign = TRUE, overparameterize = FALSE)

pred_lowSA <- predict(object = fit_lowSA, data = dl_lowSA$data, errorCon = c("RMSE","R2"), errorCat = c("CC","nCC"))

R2_lowSA <-pred_lowSA$errors

**# Plot network structure of the subgroups with highSA and lowSA #**

# Grouping variables #

col <- c("Motivation and pleasure Scale","Motivation and pleasure Scale","Motivation and pleasure Scale","Motivation and pleasure Scale","Toronto Alexithymia Scale","Toronto Alexithymia Scale","Toronto Alexithymia Scale","Social functioning")

**# Plot network structure of the subgroups with highSA #**

pdf("Network_highSA.pdf", width = 5.5,height = 5.8, family='Times', pointsize = 14)

plot_highSA <- plot(network_highSA, layout = 'circle', groups = col, color = c("#E8C32EFF","#7FBC41","#DFFFFFFF"), theme = "Borkulo", pie = pred_highSA$errors$R2, pieColor=rep('#377EB8',8), border.color = "black", legend.cex = 0.3,layoutOffset = c(-0.04,0), layoutScale = c(1.05,1.0), labels = colnames(highSA), vsize = 7, label.cex = 1.2, label.scale.equal = TRUE,label.scale = TRUE,edge.labels = TRUE,edge.label.cex=0.8)

dev.off()

**# Plot network structure of the subgroups with lowSA #**

pdf("Network_lowSA.pdf",width = 5.5,height = 5.8, family='Times', pointsize = 14)

plot_lowSA <- plot(network_lowSA, layout = 'circle', groups = col, color = c("#E8C32EFF","#7FBC41","#DFFFFFFF"), theme = "Borkulo", details = TRUE, pie = pred_lowSA$errors$R2, pieColor=rep('#377EB8',8), border.color = "black", legend.cex = 0.3, layoutOffset = c(-0.04,0), layoutScale = c(1.05,1.0), labels = colnames(lowSA), vsize = 7, label.cex = 1.2, label.scale.equal = TRUE, label.scale = TRUE, edge.labels = TRUE, edge.label.cex=0.8, edge.label.position = 0.56)

dev.off()

###########################################################################

**# 5 Gender difference in overall network structure with six dimensions of social functioning #**

###########################################################################

# read male data (n = 607) and female data (n = 2282) #

Male <- read.csv('Male.csv', header = TRUE)

Female <- read.csv('Female.csv', header = TRUE)

# rename each variable #

names(Male) <- c("Social", "Recreational", "Relationship", "Motivation", "DIF", "DDF", "EOT", "FESFS")

names(Female) <- c("Social", "Recreational", "Relationship", "Motivation", "DIF", "DDF", "EOT", "FESFS")

# Estimate the network of each group #

network_Male <- estimateNetwork(Male, default = 'EBICglasso', tuning = 0.5)

network_Female <- estimateNetwork(Female, default = 'EBICglasso', tuning = 0.5)

**# Network comparison controlling for multiple edges (Results shown in the Supplementary results) #**

mynct_gender<- NCT(network_Male,network_Female,it=1000,weighted = TRUE, test.edges = TRUE,edges = "ALL", test.centrality = TRUE, p.adjust.methods = "holm")

summary(mynct_gender)
